# Effectiveness of acupuncture for pain relief in shoulder-hand syndrome after stroke: a systematic evaluation and Bayesian network meta-analysis

**DOI:** 10.3389/fneur.2023.1268626

**Published:** 2023-11-17

**Authors:** Ting Huang, Hongfang Yao, Junneng Huang, Ning Wang, Chunjun Zhou, Xuyang Huang, Xiangyuan Tan, Yanyan Li, Yuyu Jie, Xiang Wang, Yu Yang, Yingye Liang, Siqian Yue, Yawen Mao, Songxian Lai, Jingyiqi Zheng, Yufeng He

**Affiliations:** ^1^The First School of Clinical Medicine, Guangxi University of Traditional Chinese Medicine, Nanning, China; ^2^Department of Rehabilitation Medicine, The First Affiliated Hospital of Guangxi Medical University, Nanning, China; ^3^The First Affiliated Hospital of Guangxi University of Traditional Chinese Medicine, Nanning, China; ^4^Department of Traditional Chinese Medicine, Nanning Maternal and Child Health Hospital, Nanning, China; ^5^Sainz College of New Medicine, Guangxi University of Traditional Chinese Medicine, Nanning, China

**Keywords:** acupuncture, post-stroke shoulder-hand syndrome, pain relief, systematic review, Bayesian network meta-analysis

## Abstract

**Background:**

Shoulder-hand syndrome (SHS) is a common complication after stroke, and SHS-induced pain significantly hampers patients’ overall recovery. As an alternative therapy for pain relief, acupuncture has certain advantages in alleviating pain caused by SHS after stroke. However, choosing the best treatment plan from a variety of acupuncture options is still a serious challenge in clinical practice. Therefore, we conducted this Bayesian network meta-analysis to comprehensively compare the effectiveness of various acupuncture treatment methods.

**Methods:**

We systematically searched for randomized controlled trials (RCTs) of acupuncture treatment in patients with post-stroke SHS published in PubMed, Embase, Cochrane, and Web of Science until 9 March 2023. We used the Cochrane bias risk assessment tool to assess the bias risk in the included original studies.

**Results:**

A total of 50 RCTs involving 3,999 subjects were included, comprising 19 types of effective acupuncture interventions. Compared to single rehabilitation training, the top three interventions for VAS improvement were floating needle [VAS = −2.54 (95% CI: −4.37 to −0.69)], rehabilitation + catgut embedding [VAS = −2.51 (95% CI: −4.33 to −0.68)], and other multi-needle acupuncture combinations [VAS = −2.32 (95% CI: −3.68 to −0.94)]. The top three interventions for improving the Fugl–Meyer score were eye acupuncture [Meyer = 15.73 (95% CI: 3.4627.95)], other multi-needle acupuncture combinations [Meyer = 12.22 (95% CI: 5.1919.34)], and traditional western medicine + acupuncture + traditional Chinese medicine [Meyer = 11.96 (95% CI: −0.59 to 24.63)].

**Conclusion:**

Multiple acupuncture methods are significantly effective in improving pain and upper limb motor function in post-stroke SHS, with relatively few adverse events; thus, acupuncture can be promoted.

**Systematic Review Registration:**

https://www.crd.york.ac.uk/prospero/, CRD42023410957.

## 1 Introduction

Stroke is a leading cause of disability in old people. The absolute number of stroke events worldwide increased by 70.0% from 1990 to 2019, making stroke the third leading cause of disability-adjusted life years (DALYs) (1, 2). Despite early treatment, patients with strokes often have residual sequelae, mainly characterized by hemiparesis. Shoulder hand syndrome (SHS) is a common complication after stroke, with main clinical manifestations including severe pain in the affected shoulder, along nerve distributions and injury regions, hand swelling, and sensory impairment. The incidence rate of SHS is approximately 2%–50%, with a peak incidence at 1–6 months after stroke (3, 4). Hence, this period is critical for the recovery of patients. Severe pain significantly undermines patients’ motivation for recovery, which is detrimental to the expected recovery and their return to society and family, thereby imposing a huge burden on clinical medical work (5).

Currently, treatments for post-stroke SHS pain mainly include oral medication, ganglion radiofrequency ablation, and nerve block. Oral medication can cause adverse reactions such as fatigue, liver and kidney damage, and gastrointestinal dysfunction (6–8). Nerve radiofrequency ablation and nerve block have certain analgesic effects, but as more invasive treatments, they only provide temporary pain relief and patients cannot benefit from them in the medium to long term (9). Therefore, optimizing treatment strategies for post-stroke SHS is a focus of clinical physicians. Chinese medicine acupuncture, through its unique theory of meridians, plays a positive role in treating pain, arthritis, and recovery after stroke. It is safe and effective with few adverse reactions, and it has been internationally recognized as an effective supplement and alternative therapy for pain treatment (10–12).

There are many types of acupuncture, including electroacupuncture, fire needling, warming needles, and catgut embedding. These diversified acupuncture methods have been introduced by some researchers into the treatment of post-stroke SHS, even in combination with other treatments (13). However, selecting the best acupuncture plan in clinical practice remains challenging. Therefore, we conducted this Bayesian network meta-analysis on acupuncture treatment for post-stroke SHS in the hope of providing the best acupuncture combination regimen and basis to assist in medical decision-making.

## 2 Methods

### 2.1 Study registration

This network meta-analysis was conducted based on the preferred reporting items for systematic reviews and meta-analyses (PRISMA-NMA) (14). This study has been registered on PROSPERO, and the registration website is https://www.crd.york.ac.uk/prospero/, (ID: CRD42023410957).

### 2.2 Eligibility criteria

#### 2.2.1 Inclusion criteria

P (population): patients meeting the diagnostic criteria for post-stroke SHS, regardless of age, race, and gender. Post-stroke SHS is diagnosed according to the “Rehabilitation Evaluation and Treatment of Stroke” and the “2016 China Stroke Diagnosis and Treatment Consensus” (14, 15).

The diagnostic criteria of stroke were formulated according to the “2016 China Stroke Diagnosis and Treatment Consensus.” Diagnostic points are as follows: ① acute onset; ② focal neurological deficit (such as weakness or numbness of one side of face or limb, language disorder); a few patients have comprehensive neurological deficit, which may be accompanied by headache, vomiting, elevated blood pressure, and different degrees of consciousness disorder; ③ symptoms or signs of unlimited duration (when imaging shows responsible lesions) or lasting more than 24 h (when imaging shows no responsible lesions); ④ stroke diagnosed by CT or MRI of the head, with non-cerebral vascular causes excluded.

The diagnostic criteria for shoulder-hand syndrome after stroke were the criteria established by “Rehabilitation Evaluation and Treatment of Stroke.” The diagnosis points are as follows: ① patients have unilateral shoulder and hand pain, skin flushing, skin temperature rising, and finger bending limitation after stroke. ② No evidence of local trauma, infection, and peripheral vascular disease.

I (intervention): studies that use acupuncture in the treatment group.

C (comparison): studies that use rehabilitation, medication, or conventional acupuncture in the control group.

O (outcome): the primary outcome measures are the visual analog scale (VAS) for pain and the Fugl–Meyer motor function assessment scale; the secondary outcome measures are the Barthel index (BI) and shoulder-hand syndrome evaluation scale (SHSS).

S (study design): randomized controlled trials (RCTs).

#### 2.2.2 Exclusion criteria

P (population): studies with severe problems in the diagnostic criteria for post-stroke SHS or SHS caused by other diseases.

I (intervention): (1) studies analyzing the effects of different frequencies and intensities of an intervention method, which are isolated from other intervention methods and cannot be used to estimate the effect size and (2) studies that do not include acupuncture in any of the groups.

C (comparison): none.

O (outcome): studies with serious bias in outcome measure scoring (such as those without baseline assessment or incomplete outcome data).

S (study design): (1) full conference abstracts not published through peer review; (2) studies with a sample size of <10, which are unlikely to achieve ideal statistical power; (3) crossover design studies. As the crossover design can generally achieve the preset statistical power with only a small sample size, we can only extract the results of the first phase of the trial in a systematic review. However, some crossover design studies have a small sample size and do not report the independent results of the first phase, so these studies need to be excluded.

### 2.3 Data sources and search strategy

RCTs on acupuncture treatment for post-stroke SHS are retrieved from PubMed, Cochrane, Embase, Web of Science, VIP (Chinese), Wanfang (Chinese), CNKI (Chinese), and Chinese Biomedical (Chinese) databases. There are no restrictions on language and publication years, and the retrieval date is until 12 March 2023. The retrieval is conducted in the form of “subject word + free word,” the search terms for the Chinese databases include “stroke,” “shoulder-hand syndrome,” “painful malnutrition syndrome,” “painful nutritional disorder,” “complex regional pain syndrome,” “reflex sympathetic dystrophy syndrome,” “acupuncture,” “electroacupuncture,” “warming needle,” “fire needling,” “floating needle,” and “catgut embedding “. The search strategy for the English databases is shown in Supplementary Table S1.

### 2.4 Study selection

The retrieved studies were imported into EndNote, and duplicate publications were excluded. Then, the titles and abstract were checked to preliminarily select the original studies that met the criteria and download the full text. The full texts were read to select the original RCT studies that met the criteria of this systematic review. The study selection was independently conducted by two researchers (XT and XH), and any disputes were arbitrated by a third researcher (HY) after the search was completed.

### 2.5 Data extraction

Data extraction was independently performed by two researchers (NW and YJ), and a third researcher (JH) was asked to arbitrate disputes, if any. A data extraction electronic spreadsheet was developed to extract data, including the title, English abstract, first author, year of publication, study design, author’s country, diagnostic criteria, interventional protocol, type of stroke, treatment time, number of cases, gender, age, disease course, follow-up time, outcome measures, selection bias, implementation bias, measurement bias, loss to follow-up bias, reporting bias, and other biases.

### 2.6 Assessment of study quality/risk of bias in studies

Quality assessment was conducted by two researchers (JH and XH) using Cochrane’s bias risk assessment tool RoB 2.0 (16, 17) to assess the risk of bias in the 50 included original studies. The assessment tool includes the following seven items: randomization process, method of identifying or recruiting participants, bias due to deviation from the expected intervention, missing outcome data, bias in outcome measurement, reporting selection bias, and overall bias. The risk of bias in each domain is classified into three levels: low risk of bias, high risk of bias, and some concerns. After completion, a cross-check would be conducted, and if there was a dispute, a third researcher (TH) was asked to assist in adjudicating. Finally, the risk of bias graph was plotted.

### 2.7 Outcomes

The primary outcome measures are the visual analog scale (VAS) for pain and the Fugl–Meyer upper limb motor function assessment scale. The secondary outcome measures are the Barthel index (BI) and the shoulder-hand syndrome evaluation scale (SHSS). Since our outcome measures are all continuous variables, and there seem to be differences among the studies at baseline, we used the change from baseline to post-treatment as the effect size in the meta-analysis.

### 2.8 Synthesis methods

This study used a Bayesian random-effects model to compare the effect estimates of various to compare their effectiveness. The Markov chain Monte Carlo method was used for modeling, with four Markov chains running simultaneously, and the annealing times were set to 20,000. The modeling was completed after 50,000 simulation iterations. The deviance information criterion (DIC) was used to compare model fit and global consistency. If there is a closed loop in the network, we would use the node-splitting method to analyze local consistency. In addition, we ranked these interventions based on the surface under the cumulative ranking curve (SUCRA) values, and a league table was generated to compare the differences in effectiveness between various interventions. A funnel plot was used to intuitively reflect the heterogeneity among the studies. Analyses were completed in Stata 15.0 (Stata Corporation, College Station, TX) and R 4.2.0 (R Development Core Team, Vienna, http://www.R-project.org). A *p*-value of <0.05 indicates a statistically significant difference.

## 3 Results

### 3.1 Study selection

**Figure 1:**
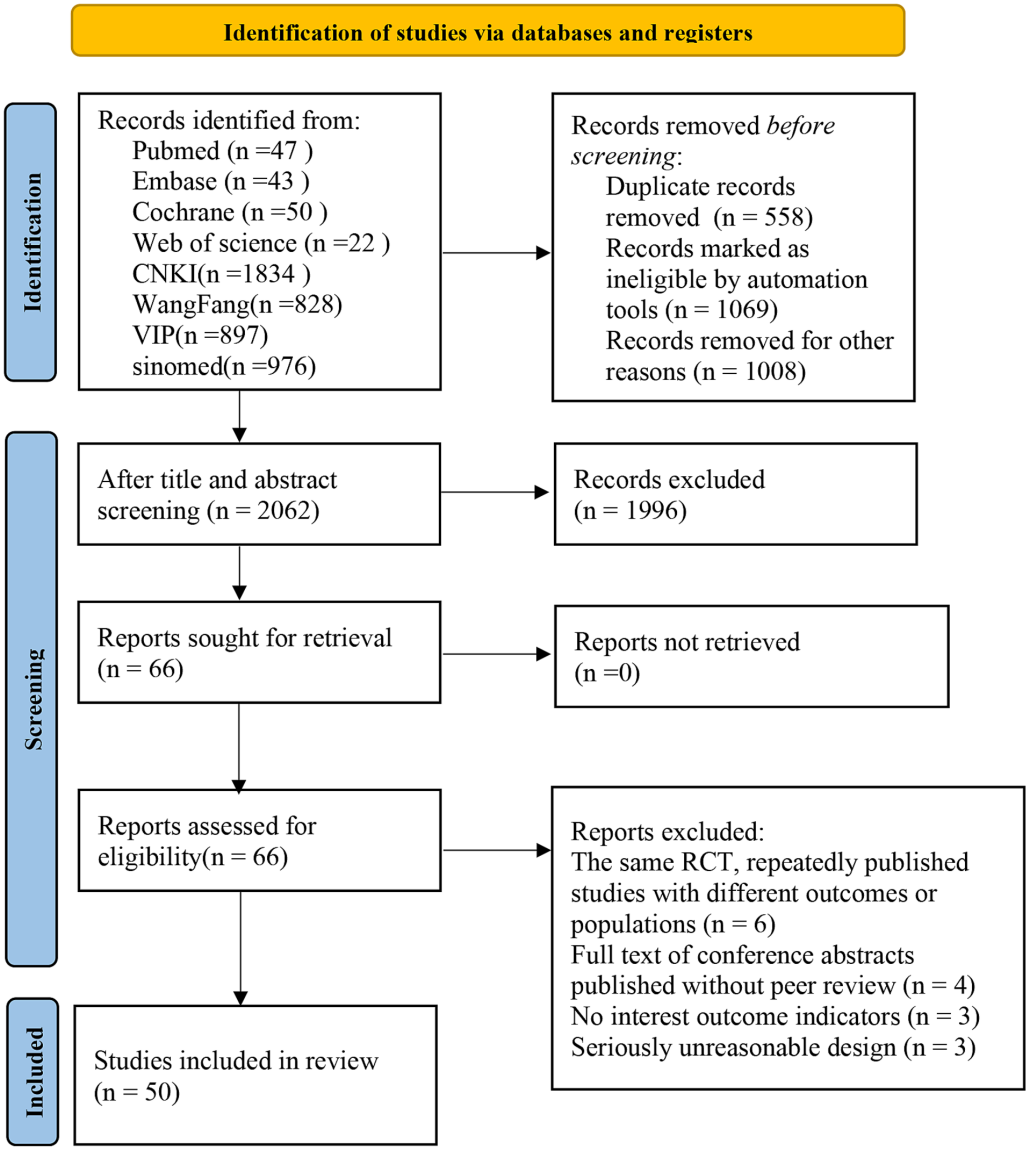
Literature screening flow chart.

A total of 4,697 relevant articles were retrieved from the databases, including 162 from English databases and 4,535 from Chinese databases. A total of 558 duplicate articles were marked in EndNote and eliminated. After reading the titles and abstracts of the remaining 4,139 articles, we finally determined to download the full texts of 66 articles. According to a full-text review, 50 RCTs (18–67) were eligible and included in this systematic review (as shown in Figure 1; Supplementary Table S2).

### 3.2 Study characteristics

This study included 50 articles in total, involving 3,999 patients with SHS after stroke, and 2 of them were multi-center RCTs (41, 42). The included articles were published between 2002 and 2022, covering 19 acupuncture treatment methods. There were 11 types of acupuncture combined with other treatments. None of the original studies we included had conflicts of interest. The basic information of the included studies can be found in Table 1.

**Table 1:** Basic information of the included studies.

No.	First author	Year of publication	Study design	Author’s country	Post-stroke SHS	Interventional protocol	Type of Ssroke (hemorrhage/ischemia)	Treatment time	Number of cases	Total population	Gender (M/F)	Age	Disease course
1	Ying Wang	2002	Single-center	China	Rehabilitation Assessment and Treatment of Stroke	Treat 12 (special needle operation and technique) Treat 4 (rehabilitation + acupuncture)	None	8w	83 52	135	48/35 30/22	62.32 (mean) 62.32 (mean)	45 (d) 45 (d)
2	Yongqiang He	2022	Single-center	China	Diagnosis, Treatment and Consensus of Cerebrovascular Diseases in China (2016 Edition)	Treat 6 (electroacupuncture) Treat 14 (acupuncture on the non-affected limb)	None	None	40 40	80	24/16 26/14	34–80 35–78	15 (d)–4 (m) 15 (d)–4 (m)
3	Xinru Li	2021	Single-center	China	Stroke Rehabilitation	Treat 1 (rehabilitation) Treat 4 (rehabilitation + acupuncture) Treat 4 (rehabilitation + acupuncture) Treat 7 (warm acupuncture)	No description	4w	24 24 24 24	96	19/5 18/6 15/9 17/7	60.79 ± 12.06 67.54 ± 11.35 63.74 ± 12.45 65.74 ± 12.17	29.14 ± 5.34 (d) 30.45 ± 5.76 (d) 29.52 ± 5.35 (d) 30.24 ± 5.86 (d)
4	Juan Yang	2011	Single-center	China	Rehabilitation Assessment and Treatment of Stroke	Treat 8 (fire needling) Treat 5 (western medicine + rehabilitation + acupuncture)	13/18 12/19	2w	31 31	62	17/14 18/13	60.85 ± 8.80 63.12 ± 8.33	None
5	Qin Xie	2011	Single-center	China	Rehabilitation Assessment and Treatment of Stroke	Treat 9 (Jin’s three-needle technique) Treat 1 (rehabilitation)	0/60	4w	30 30	60	13/17 14/16	64.83 ± 8.81 63.80 ± 9.81	None
6	Xiaoqiong Hua	2022	Single-center	China	Rehabilitation Assessment and Treatment of Stroke	Treat 2 (rehabilitation + western medicine) Treat 5 (western medicine + rehabilitation + acupuncture)	None	4w	52 53	105	None	18–75 18–75	15–90 (d) 15–90 (d)
7	Liang Zhou	2016	Single-center	China	No description	Treat 14 (acupuncture on the non-affected limb) Treat 4 (rehabilitation + acupuncture)	None	4w	20 20	40	12/8 11/9	55.9 ± 11.2 56.3 ± 10.9	27.3 ± 9.6 (d) 29.6 ± 8.7 (d)
8	Feilin Ni	2021	Single-center	China	Neurological Rehabilitation	Treat 2 (rehabilitation + western medicine) Treat 5 (western medicine + rehabilitation + acupuncture)	0/63	4w	31 32	63	9/22 11/21	67.2 ± 6.5 68.3 ± 8.5	No description
9	Qingbo Ju	2016	Single-center	China	Rehabilitation Assessment and Treatment of Stroke	Treat 11 (eye acupuncture) Treat 1 (rehabilitation)	No description	4w	34 34	68	20/14 18/15	54.23 ± 9.37 56.37 ± 10.88	58 ± 10.42 (d) 55 ± 7.34 (d)
10	Wenjun Nong	2012	Single-center	China	Rehabilitation Assessment and Treatment of Stroke	Treat 13 (other multi-needle acupuncture combinations) Treat 4 (rehabilitation + acupuncture)	No description	3w	40 40	80	22/18 25/15	63 ± 7.50 61 ± 9.60	48 ± 9.60 (d) 46 ± 9.80 (d)
11	Yimo Feng	2015	Single-center	China	Clinical Rehabilitation Medicine	Treat 1 (rehabilitation) Treat 4 (rehabilitation + acupuncture)	No description	4w	57 69	126	83/43	63.4 ± 5.3 63.4 ± 5.3	33.1 ± 6.7 (d) 33.1 ± 6.7 (d)
12	Xianping Huang	2017	Single-center	China	No description	Treat 1 (rehabilitation) Treat 15 (western medicine + acupuncture + Chinese medicine)	29/36 30/35	3w	65 65	130	34/31 33/32	62.16 ± 5.27 62.12 ± 5.31	40.26 ± 7.24 (d) 40.21 ± 7.27 (d)
13	Ranwei Li	2020	Single-center	China	Rehabilitation Assessment and Treatment of Stroke	Treat 5 (western medicine + rehabilitation + acupuncture) Treat 14 (acupuncture on the non-affected limb)	0/62	4w	31 31	62	17/14 18/13	57.7 ± 7.4 58.2 ± 6.7	1.2 ± 0.3 (m) 1.3 ± 0.3 (m)
14	Lijuan Cao	2020	Single-center	China	Neurological Rehabilitation	Treat 5 (western medicine + rehabilitation + acupuncture) Treat 13 (other multi-needle acupuncture combinations)	No description	3w	39 39	78	30/9 28/11	61.4 ± 9.8 60.1 ± 10.7	39.5 ± 24.7 (d) 41.3 ± 23.2 (d)
15	Ronghua Zhu	2018	Single-center	China	Rehabilitation Assessment and Treatment of Stroke	Treat 16 (rehabilitation + catgut embedding) Treat 2 (rehabilitation + western medicine) Treat 16 (rehabilitation + catgut embedding)	6/24 5 out of 25 5 out of 25	6w	30 30 30	90	21/9 20/10 21/9	61.6 ± 10.3 59.9 ± 8.7 62.8 ± 7.3	56.3 ± 15.7 (d) 62.7 ± 14.2 (d) 59.1 ± 12.2 (d)
16	Xiongjie Chen	2015	Single-center	China	Clinical Rehabilitation Medicine	Treat 14 (acupuncture on the non-affected limb) Treat 6 (electroacupuncture) Treat 18 (gangliolysis)	No description	2w	20 20 20	60	No description	42–70 42–70 42–70	1–6 (m) 1–6 (m)
17	Zouqin Huang	2015	Multi-center	China	Rehabilitation Assessment and Treatment of Stroke	Treat 6 (electroacupuncture) Treat 1 (rehabilitation)	No description	8w	30 30	60	21/9 20/10	61 ± 15 62 ± 8	34.93 ± 17.83 (d) 35.48 ± 20.06 (d)
18	Miaojun Lin	2016	Single-center	China	No description	Treat 16 (rehabilitation + catgut embedding) Treat 5 (western medicine + rehabilitation + acupuncture)	24/36 26/34	5w	60 60	120	32/28 27/33	63 ± 4 64 ± 4	7.02 ± 4.03 (m) 8.34 ± 4.29 (m)
19	Liwen Xue	2007	No description	China	No description	Treat 12 (special needle operation and technique) Treat 6 (electroacupuncture)	5/35 3/37	4w	40 40	80	25/15 23/17	61.11 ± 7.12 60.72 ± 7.01	1–56 (d) 1–53 (d)
20	Ruiqing Li	2022	Single-center	China	Chinese Standards for Diagnosis and Treatment of Rehabilitation Medicine (Volume 2)	Treat 13 (other multi-needle acupuncture combinations) Treat 5 (western medicine + rehabilitation + acupuncture)	15/17 12/20	3w	32 32	64	18/14 15/17	49 ± 2 54 ± 1	139.6 ± 2.7 (d) 138.9 ± 2.2 (d)
21	Yanjie Shang	2008	Single-center	China	Rehabilitation Assessment and Treatment of Stroke	Treat 4 (rehabilitation + acupuncture) Treat 3 (routine acupuncture) Treat 1 (rehabilitation)	No description	5w	40 40 40	120	28/12 27/13 25/15	53.42 ± 6.17 52.38 ± 6.25 51.79 ± 6.14	5.23 ± 1.47 (m) 5.37 ± 1.42 (m) 5.02 ± 1.38 (m)
22	Jie Zhan	2022	Single-center	China	Guidelines and Consensus on the Diagnosis and Treatment of Cerebrovascular Diseases of the Chinese Society of Neurology	Treat 14 (acupuncture on the non-affected limb) Treat 1 (rehabilitation)	9/16 7/17	2w	25 24	49	16/9 14/10	59.36 ± 8.73 55.50 ± 8.20	59.64 ± 31.07 (d) 68.17 ± 41.09 (d)
23	Fanying Meng	2014	Single-center	China	Stroke Rehabilitation	Treat 4 (rehabilitation + acupuncture) Treat 7 (warm acupuncture)	No description	2w	30 30	60	17/13 16/14	69.3 ± 5.7 68.7 ± 5.2	19.8 ± 3.7 (d) 19.0 ± 2.9 (d)
24	Xiaoli Tang	2021	Single-center	China	Guidelines and Consensus on the Diagnosis and Treatment of Cerebrovascular Diseases in China (2016 Edition)	Treat 7 (warm acupuncture) Treat 1 (rehabilitation)	12/24 11/25	4w	36 36	72	21/15 20/16	69.3 ± 7.1 68.9 ± 6.8	33.08 ± 9.54 (d) 32.64 ± 9.22 (d)
25	Jun Wang	2013	Single-center	China	Neurological Rehabilitation	Treat 10 (floating needle) Treat 2 (rehabilitation + western medicine) Treat 17 (western medicine + rehabilitation + Chinese medicine)	12/18 14/16 18/12	4w	30 30 30	90	16/14 13/17 15/15	61.4 ± 9.7 61.9 ± 9.8 63.2 ± 10.3	3w–6 (m) 3w–5 (m) 3w–6 (m)
26	Jinbiao Hong	2009	Single-center	China	Rehabilitation Assessment and Treatment of Stroke	Treat 14 (acupuncture on the non-affected limb) Treat 3 (routine acupuncture)	8/22 7/23	4w	30 30	60	14/16 17/13	60.20 ± 9.06 61.50 ± 9.32	31.30 ± 3.11 (d) 31.87 ± 3.30 (d)
27	Qian Zhang	2015	Single-center	China	Chinese Rehabilitation Medicine Diagnosis and Treatment Standards	Treat 12 (special needle operation and technique) Treat 5 (western medicine + rehabilitation + acupuncture)	0/60	4w	30 30	60	17/13 19/11	62 ± 9 62 ± 9	49.3 ± 8.6 (d) 51.2 ± 9.4 (d)
28	Bing Yan	2015	Single-center	China	Rehabilitation Assessment and Treatment of Stroke	Treat 9 (Jin’s three-needle technique) Treat 3 (routine acupuncture)	13/17 9/21	2w	30 30	60	16/14 13/17	41–85 35–85	15 d–153 (d) 17 d–158 (d)
29	Tongbo Jiang	2016	Single-center	China	No description	Treat 12 (special needle operation and technique) Treat 3 (routine acupuncture)	0/60	3w	30 31	60	18/12 19/11	62 (mean) 58 (mean)	50 (d) 55 (d)
30	Chunshui Huang	2017	Single-center	China	Neurological Rehabilitation	Treat 4 (rehabilitation + acupuncture) Treat 1 (rehabilitation)	11/19 8/22	3w	30 30	60	43/17	59 ± 5 59 ± 6	46.8 ± 14.8 (d) 45.4 ± 16.5 (d)
31	Zhaohui Zhou	2014	Single-center	China	Rehabilitation Assessment and Treatment of Stroke	Treat 10 (floating needle) Treat 4 (rehabilitation + acupuncture)	37/13 41/9	2w	50 50	100	26/24 23/27	65 ± 9 66 ± 12	62.2 ± 42.5 (d) 63.7 ± 44.4 (d)
32	Wenrong Wan	2013	Single-center	China	Rehabilitation Assessment and Treatment of Stroke	Treat 1 (rehabilitation) Treat 4 (rehabilitation + acupuncture)	56/64	4w	60 60	120	34/26 32/28	63 ± 6 60 ± 6	33.0 ± 9.4 (d) 38.4 ± 9.0 (d)
33	Bingfeng Xing	2019	Single-center	China	Neurological Rehabilitation	Treat 4 (rehabilitation + acupuncture) Treat 16 (rehabilitation + catgut embedding)	10/19/1 9/20/1	4w	30 30	60	16/14 17/13	54.1 ± 9.1 55.8 ± 8.8	3.1 ± 1.2 (m) 3.3 ± 1.0 (m)
34	Ning Li	2012	Multi-center	China	Chinese Rehabilitation Diagnosis and Treatment Standards	Treat 6 (electroacupuncture) Treat 1 (rehabilitation)	No description	6w	60 60	120	20/40 19/41	62 ± 12 61 ± 13	28 ± 6 (d) 27 ± 5 (d)
35	Jingchun Yin	2015	Single-center	China	Rehabilitation Assessment and Treatment of Stroke	Treat 13 (other multi-needle acupuncture combinations) Treat 1 (rehabilitation)	5 out of 25 5 out of 25	4w	30 30	60	20/10 19/11	63 ± 9 62 ± 10	67.17 ± 14.50 (d) 67.10 ± 15.21 (d)
36	Jingjun Xie	2016	Single-center	China	No description	Treat 6 (electroacupuncture) Treat 2 (rehabilitation + western medicine)	0/80	4w	40 40	80	27/13 22/18	50 ± 11 51 ± 10	14d–2 (m) 15d–2 (m)
37	Sen Gao	2022	Single-center	China	Neurological Rehabilitation	Treat 5 (western medicine + rehabilitation + acupuncture) Treat 12 (special needle operation and technique)	11/39 9/42	8w	50 51	106	28/22 30/21	61 ± 11 63 ± 12	60 ± 24 (d) 58 ± 22 (d)
38	Zhihong Zou	2021	Single-center	China	Stroke Rehabilitation	Treat 12 (special needle operation and technique) Treat 5 (western medicine + rehabilitation + acupuncture)	25/32 29/26	3w	55 57	112	34/23 28/27	55.74 ± 8.56 56.02 ± 8.69	49.35 ± 19.56 (d) 51.31 ± 20.24 (d)
39	Xueping Yu	2017	Single-center	China	Rehabilitation Assessment and Treatment of Stroke	Treat 12 (special needle operation and technique) Treat 3 (routine acupuncture)	12/17 15/13	2w	29 28	57	19/10 17/11	56.48 ± 8.53 56.39 ± 8.72	51.13 ± 24.82 (d) 51.54 ± 24.07 (d)
40	Shurong Wang	2022	Single-center	China	Rehabilitation Assessment and Treatment of Stroke	Treat 13 (other multi-needle acupuncture combinations) Treat 5 (western medicine + rehabilitation + acupuncture)	No description	4w	30 30	60	16/14 15/15	54.83 ± 4.58 54.90 ± 3.49	55.80 ± 25.66 (d) 55.33 ± 20.99 (d)
41	Mingming Wang	2018	Single-center	China	Stroke Rehabilitation	Treat 6 (electroacupuncture) Treat 3 (routine acupuncture)	No description	4w	43 43	86	24/19 23/20	56.9 ± 10.3 58.1 ± 9.8	20.3 ± 4.5 (d) 19. 8 ± 3.7 (d)
42	Weidong Yang	2019	Single-center	China	Rehabilitation Assessment and Treatment of Stroke	Treat 2 (rehabilitation + western medicine) Treat 6 (electroacupuncture)	No description	4w	30 30	60	18/12 20/10	54–75 51–72	38 ± 11 (d) 35 ± 9 (d)
43	Yang Wang	2019	Single-center	China	Rehabilitation Assessment and Treatment of Stroke	Treat 12 (special needle operation and technique) Treat 6 (electroacupuncture)	No description	5w	36 36	72	19/17 16/20	66.7 ± 11.3 65.8 ± 14.5	38.2 ± 16.8 (d) 37.9 ± 19.1 (d)
44	Shengsan He	2016	Single-center	China	Rehabilitation Assessment and Treatment of Stroke	Treat 14 (acupuncture on the non-affected limb) Treat 1 (rehabilitation)	36/24 34/26	3w	60 60	120	34/26 32/28	50.66 ± 10.24 48.12 ± 11.98	21.07 ± 8.69 (d) 20.32 ± 8.61 (d)
45	Jingchang Huang	2013	Single-center	China	Rehabilitation Assessment and Treatment of Stroke	Treat 8 (fire needling) Treat 3 (routine acupuncture)	0/70	3w 2w	35 35	70	19/16 18/17	62 ± 7 60 ± 7	<2 (m)
46	Youhua Zeng	2019	Single-center	China	Chinese Rehabilitation Medicine Diagnosis and Treatment Standards	Treat 13 (other multi-needle acupuncture combinations) Treat 4 (rehabilitation + acupuncture)		4w	56 56	112	29/27 30/26	68 ± 12 69 ± 9	30–99 (d) 15–102 (d)
47	Ting Zhang	2019	Single-center	China	Rehabilitation Assessment and Treatment of Stroke	Treat 12 (special needle operation and technique) Treat 1 (rehabilitation)	No description	4w	31 31	62	18/13 19/12	53.2 ± 11.2 54.1 ± 10.4	72.3 ± 17.1 (d) 74.9 ± 15.2 (d)
48	Fei Huang	2018	Single-center	China	Rehabilitation Medicine for Stroke	Treat 13 (other multi-needle acupuncture combinations) Treat 6 (electroacupuncture)	No description	2w	30 30	60	18/12 20/10	50.7 ± 11.2 52.3 ± 9.8	152.8 ± 99.2 (d) 147.6 ± 85.7 (d)
49	Guoqi Chen	2018	Single-center	China	Rehabilitation Assessment and Treatment of Stroke	Treat 14 (acupuncture on the non-affected limb) Treat 5 (western medicine + rehabilitation + acupuncture)	No description	2w	36 36	72	19/17 18/18	51.5 ± 7.9 53.2 ± 7.6	64.6 ± 39.8 (d) 65.8 ± 38.9 (d)
50	Xin Tong	2014	Single-center	China	Rehabilitation Assessment and Treatment of Stroke	Treat 13 (other multi-needle acupuncture combinations) Treat 3 (routine acupuncture)	0/60	3w	30 30	60	18/12 21/9	60 ± 9 59 ± 9	45.2 ± 9.6 (d) 46.7 ± 9.1 (d)

### 3.3 Detailed explanation of intervention methods

This systematic review covers 19 acupuncture treatment methods (routine acupuncture).

Point-toward-point needle insertion, warming needles, electroacupuncture, fire needling, Jin’s three-needle technique, floating needle, eye acupuncture, silver needle, trocar needle, spoon-like needle, thumbtack needle, abdominal acupuncture, scalp acupuncture, contralateral blood-letting and contralateral needling, catgut embedding, wrist-ankle acupuncture, balancing acupuncture, eightfold method of the sacred tortoise, four special acupuncture techniques (18, 44, 55, 56, 64), and four special needle insertion methods (36, 42, 46, 47, 60, 63, 66). Affected limb movement during acupuncture was reported in three studies (24, 44, 67). The detailed intervention methods are provided in Supplementary Table S3.

### 3.4 Results of bias risk assessment

**Figure 2:**
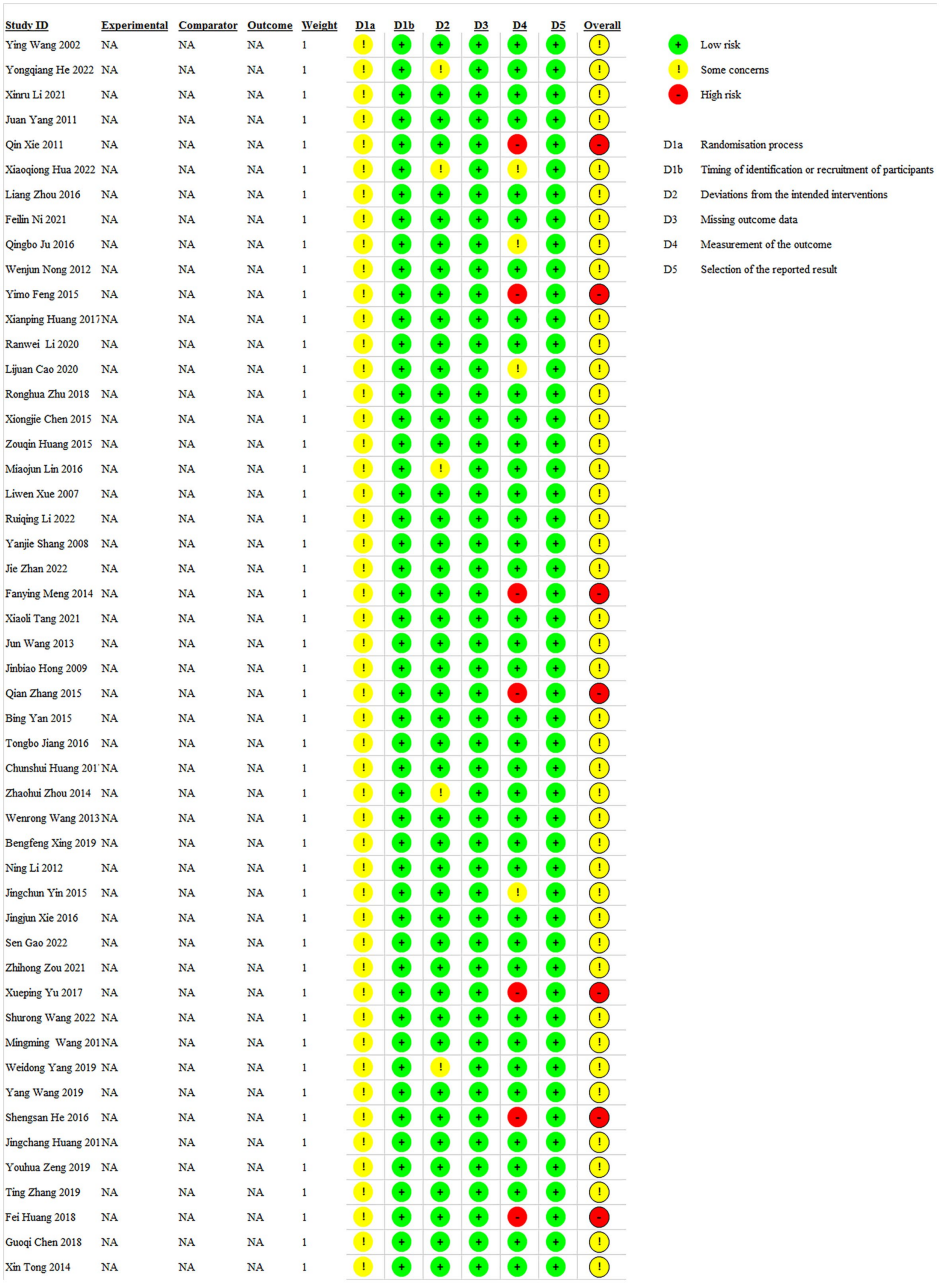
Bias risk assessment results of the included original studies.

In our 50 included studies, 43 were assessed as having “some concerns” for overall bias and 7 had a high risk of bias. The high risk of bias in the seven studies was because the intervention implementers knew the intervention. Since there was no detailed information on allocation concealment in these 50 RCTs, 43 studies were classified as “some concerns.” Due to the experimental environment, five studies deviated from the predetermined intervention measures and were assessed as having “some concerns.” During the implementation of the intervention, because the evaluators knew the intervention, seven studies were classified as high risk and four as some concerns. The detailed bias risk assessment results are presented in Figure 2.

## 4 Meta-analysis

### 4.1 VAS

#### 4.1.1 Correlation between intervention methods

**Figure 3:**
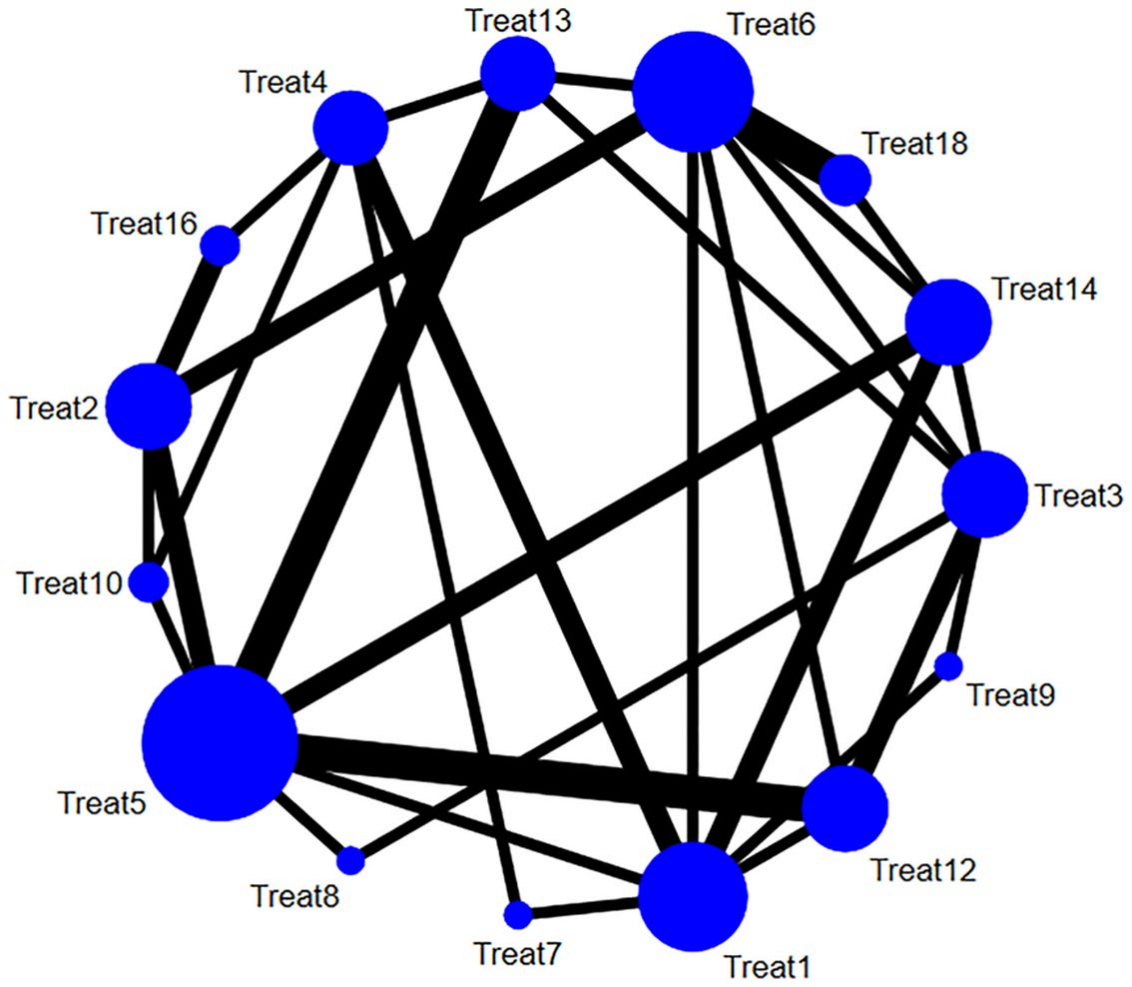
Network relationship diagram of the VAS pain relief of multiple acupuncture methods for post-stroke SHS (the larger the dot in the figure, the more samples it contains; the thicker the line between two dots, the more documents are compared between the two groups).

This review included 38 studies (19–22, 28–34, 37, 39–46, 48, 50, 52–67) that reported the pain relief effect of multiple acupuncture methods for post-stroke SHS using the VAS scale. The main intervention methods included Treat 5 (western medicine + rehabilitation + acupuncture), Treat 6 (electroacupuncture), Treat 1 (rehabilitation), Treat 3 (routine acupuncture), and Treat 14 (acupuncture on the non-affected limb). With the development of acupuncture in recent years, Treat 7 (warming needle), Treat 8 (fire needling), Treat 9 (Jin’s three-needle technique), Treat 10 (floating needle) have also gradually started to be explored for their use in pain relief in SHS after stroke. A detailed comparison of the intervention methods is illustrated in Figure 3.

#### 4.1.2 Synthesized results

**Figure 4:**
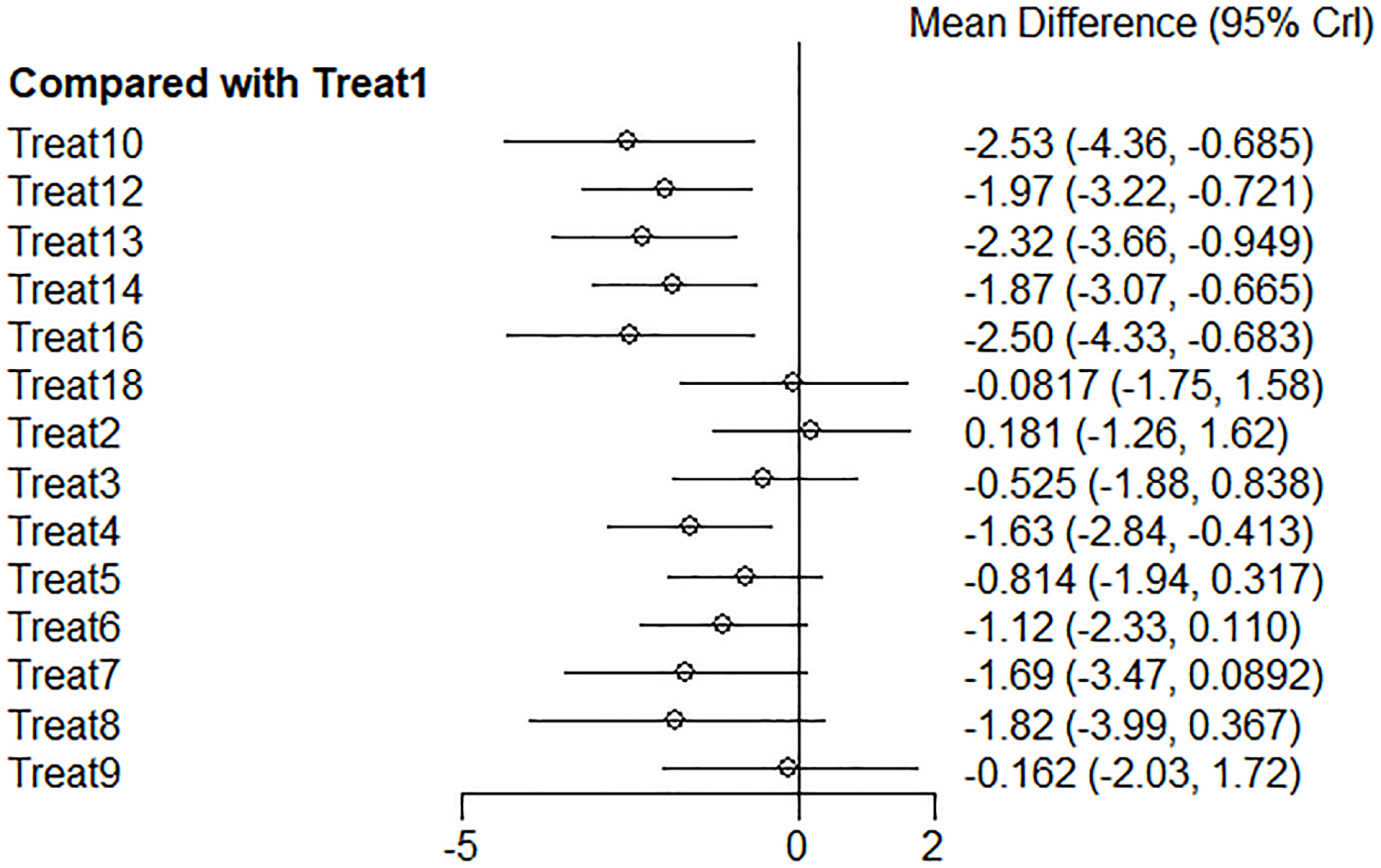
VAS forest plot of network meta-analysis of acupuncture treatments versus rehabilitation therapy for post-stroke shoulder-hand syndrome [the left side of the picture represents the results obtained after comparing various intervention methods with the conventional group Treat 1 (rehabilitation). The horizontal line for each intervention represents the confidence interval for estimating the effect size. If the confidence interval does not contain a null line, the difference is statistically significant].

The studies included in this analysis met the overall consistency hypothesis test (*p* > 0.05), so we used a consistency model for Bayesian network meta-analysis. The results of the analysis show that compared to the routine intervention Treat 1 (rehabilitation), Treat 10 (floating needle), Treat 16 (rehabilitation + catgut embedding), Treat 13 (other multi-needle acupuncture combinations), Treat 12 (special needle operation and technique), Treat 14 (acupuncture on the non-affected limb), Treat 8 (fire needling), Treat 7 (warming needle), Treat 4 (rehabilitation + acupuncture), Treat 6 (electroacupuncture), Treat 5 (western medicine + rehabilitation + acupuncture), and Treat 3 (routine acupuncture) could significantly reduce VAS score, (*p* < 0.05) as shown in Figure 4.

Among these effective intervention methods, the top three were Treat 10 (floating needle), Treat 16 (rehabilitation + catgut embedding), and Treat 13 (other multi-needle acupuncture combinations), with ranking probabilities of 0.84, 0.83, and 0.82, respectively. At the same time, compared to routine rehabilitation, their improvements in VAS were Treat 10 (floating needle) −2.54 (95% CI: −4.37 to −0.69), Treat 16 (rehabilitation + catgut embedding) −2.51 (95% CI: −4.33 to −0.68), and Treat 13 (other multi-needle acupuncture combinations) −2.32 (95% CI: −3.68 to −0.94), with significant differences. The detailed results of pairwise comparisons are shown in Table 2.

**Table 2:** Fugl–Meyer league table of network meta-analysis of acupuncture treatment for post-stroke SHS.

Treat 1	−2.54 (−4.37, −0.69)	−1.98 (−3.23, −0.7)	−2.32 (−3.68, −0.94)	−1.87 (−3.08, −0.66)	−2.51 (−4.33, −0.68)	−0.09 (−1.75, 1.59)	0.18 (−1.26, 1.62)	−0.53 (−1.89, 0.84)	−1.63 (−2.84, −0.4)	−0.82 (−1.95, 0.32)	−1.12 (−2.34, 0.12)	−1.69 (−3.46, 0.07)	−1.83 (−3.99, 0.36)	−0.16 (−2.03, 1.73)
2.54 (0.69, 4.37)	Treat 10	0.56 (−1.38, 2.5)	0.22 (−1.7, 2.13)	0.66 (−1.29, 2.6)	0.03 (−2.11, 2.16)	2.45 (0.23, 4.65)	2.71 (0.92, 4.49)	2.01 (−0.01, 4.01)	0.91 (−0.83, 2.64)	1.72 (−0.02, 3.45)	1.42 (−0.46, 3.29)	0.85 (−1.53, 3.2)	0.7 (−1.88, 3.29)	2.38 (−0.12, 4.89)
1.98 (0.7, 3.23)	−0.56 (−2.5, 1.38)	Treat 12	−0.34 (−1.71, 1.03)	0.1 (−1.25, 1.43)	−0.53 (−2.46, 1.37)	1.89 (0.19, 3.6)	2.15 (0.67, 3.63)	1.45 (0.23, 2.66)	0.35 (−1.19, 1.88)	1.15 (0.1, 2.21)	0.86 (−0.39, 2.1)	0.29 (−1.82, 2.37)	0.14 (−1.97, 2.27)	1.81 (−0.19, 3.84)
2.32 (0.94, 3.68)	−0.22 (−2.13, 1.7)	0.34 (−1.03, 1.71)	Treat 13	0.44 (−0.98, 1.85)	−0.19 (−2.1, 1.72)	2.23 (0.48, 3.99)	2.5 (0.99, 3.99)	1.79 (0.38, 3.18)	0.69 (−0.76, 2.13)	1.5 (0.4, 2.58)	1.2 (−0.11, 2.5)	0.63 (−1.48, 2.71)	0.48 (−1.68, 2.66)	2.16 (0.06, 4.27)
1.87 (0.66, 3.08)	−0.66 (−2.6, 1.29)	−0.1 (−1.43, 1.25)	−0.44 (−1.85, 0.98)	Treat 14	−0.63 (−2.55, 1.29)	1.79 (0.24, 3.34)	2.05 (0.57, 3.55)	1.35 (0, 2.69)	0.25 (−1.27, 1.77)	1.05 (−0.08, 2.2)	0.76 (−0.44, 1.96)	0.18 (−1.89, 2.25)	0.04 (−2.12, 2.24)	1.71 (−0.31, 3.77)
2.51 (0.68, 4.33)	−0.03 (−2.16, 2.11)	0.53 (−1.37, 2.46)	0.19 (−1.72, 2.1)	0.63 (−1.29, 2.55)	Treat 16	2.42 (0.27, 4.58)	2.68 (1.23, 4.15)	1.98 (−0.01, 3.97)	0.88 (−0.86, 2.63)	1.68 (−0.06, 3.45)	1.39 (−0.4, 3.19)	0.82 (−1.55, 3.16)	0.67 (−1.89, 3.26)	2.35 (−0.13, 4.84)
0.09 (−1.59, 1.75)	−2.45 (−4.65, −0.23)	−1.89 (−3.6, −0.19)	−2.23 (−3.99, −0.48)	−1.79 (−3.34, −0.24)	−2.42 (−4.58, −0.27)	Treat 18	0.26 (−1.49, 2)	−0.45 (−2.19, 1.29)	−1.54 (−3.41, 0.33)	−0.74 (−2.36, 0.88)	−1.03 (−2.32, 0.25)	−1.61 (−3.97, 0.74)	−1.75 (−4.2, 0.71)	−0.08 (−2.4, 2.27)
−0.18 (−1.62, 1.26)	−2.71 (−4.49, −0.92)	−2.15 (−3.63, −0.67)	−2.5 (−3.99, −0.99)	−2.05 (−3.55, −0.57)	−2.68 (−4.15, −1.23)	−0.26 (−2, 1.49)	Treat 2	−0.7 (−2.28, 0.86)	−1.8 (−3.32, −0.28)	−1 (−2.25, 0.25)	−1.3 (−2.56, −0.03)	−1.86 (−4.02, 0.27)	−2.01 (−4.27, 0.26)	−0.33 (−2.53, 1.86)
0.53 (−0.84, 1.89)	−2.01 (−4.01, 0.01)	−1.45 (−2.66, −0.23)	−1.79 (−3.18, −0.38)	−1.35 (−2.69, 0)	−1.98 (−3.97, 0.01)	0.45 (−1.29, 2.19)	0.7 (−0.86, 2.28)	Treat 3	−1.1 (−2.71, 0.51)	−0.29 (−1.54, 0.96)	−0.59 (−1.89, 0.71)	−1.16 (−3.31, 0.98)	−1.31 (−3.36, 0.77)	0.37 (−1.46, 2.23)
1.63 (0.4, 2.84)	−0.91 (−2.64, 0.83)	−0.35 (−1.88, 1.19)	−0.69 (−2.13, 0.76)	−0.25 (−1.77, 1.27)	−0.88 (−2.63, 0.86)	1.54 (−0.33, 3.41)	1.8 (0.28, 3.32)	1.1 (−0.51, 2.71)	Treat 4	0.8 (−0.55, 2.16)	0.51 (−0.96, 1.98)	−0.06 (−1.85, 1.7)	−0.21 (−2.51, 2.11)	1.47 (−0.68, 3.62)
0.82 (−0.32, 1.95)	−1.72 (−3.45, 0.02)	−1.15 (−2.21, −0.1)	−1.5 (−2.58, −0.4)	−1.05 (−2.2, 0.08)	−1.68 (−3.45, 0.06)	0.74 (−0.88, 2.36)	1 (−0.25, 2.25)	0.29 (−0.96, 1.54)	−0.8 (−2.16, 0.55)	Treat 5	−0.3 (−1.44, 0.84)	−0.87 (−2.88, 1.12)	−1.01 (−2.96, 0.95)	0.66 (−1.32, 2.67)
1.12 (−0.12, 2.34)	−1.42 (−3.29, 0.46)	−0.86 (−2.1, 0.39)	−1.2 (−2.5, 0.11)	−0.76 (−1.96, 0.44)	−1.39 (−3.19, 0.4)	1.03 (−0.25, 2.32)	1.3 (0.03, 2.56)	0.59 (−0.71, 1.89)	−0.51 (−1.98, 0.96)	0.3 (−0.84, 1.44)	Treat 6	−0.57 (−2.64, 1.48)	−0.72 (−2.87, 1.46)	0.96 (−1.07, 2.99)
1.69 (−0.07, 3.46)	−0.85 (−3.2, 1.53)	−0.29 (−2.37, 1.82)	−0.63 (−2.71, 1.48)	−0.18 (−2.25, 1.89)	−0.82 (−3.16, 1.55)	1.61 (−0.74, 3.97)	1.86 (−0.27, 4.02)	1.16 (−0.98, 3.31)	0.06 (−1.7, 1.85)	0.87 (−1.12, 2.88)	0.57 (−1.48, 2.64)	Treat 7	−0.15 (−2.85, 2.6)	1.53 (−0.99, 4.09)
1.83 (−0.36, 3.99)	−0.7 (−3.29, 1.88)	−0.14 (−2.27, 1.97)	−0.48 (−2.66, 1.68)	−0.04 (−2.24, 2.12)	−0.67 (−3.26, 1.89)	1.75 (−0.71, 4.2)	2.01 (−0.26, 4.27)	1.31 (−0.77, 3.36)	0.21 (−2.11, 2.51)	1.01 (−0.95, 2.96)	0.72 (−1.46, 2.87)	0.15 (−2.6, 2.85)	Treat 8	1.67 (−0.98, 4.31)
0.16 (−1.73, 2.03)	−2.38 (−4.89, 0.12)	−1.81 (−3.84, 0.19)	−2.16 (−4.27, −0.06)	−1.71 (−3.77, 0.31)	−2.35 (−4.84, 0.13)	0.08 (−2.27, 2.4)	0.33 (−1.86, 2.53)	−0.37 (−2.23, 1.46)	−1.47 (−3.62, 0.68)	−0.66 (−2.67, 1.32)	−0.96 (−2.99, 1.07)	−1.53 (−4.09, 0.99)	−1.67 (−4.31, 0.98)	Treat 9

Treat 1 (rehabilitation), Treat 2 (rehabilitation + western medicine), Treat 3 (routine acupuncture), Treat 4 (rehabilitation + acupuncture), Treat 5 (western medicine + rehabilitation + acupuncture), Treat 6 (electroacupuncture), Treat 7 (warm acupuncture), Treat 8 (fire needling), Treat 9 (Jin’s three-needle technique), Treat 10 (floating needle), Treat 11 (eye acupuncture), Treat 12 (special needle operation and technique), Treat 13 (other multi-needle acupuncture combinations), Treat 14 (acupuncture on the non-affected limb), Treat 15 (western medicine + acupuncture + Chinese medicine), Treat 16 (rehabilitation + catgut embedding), Treat 17 (western medicine + rehabilitation + Chinese medicine), Treat 18 (gangliolysis).

#### 4.1.3 Reporting biases

**Figure 5:**
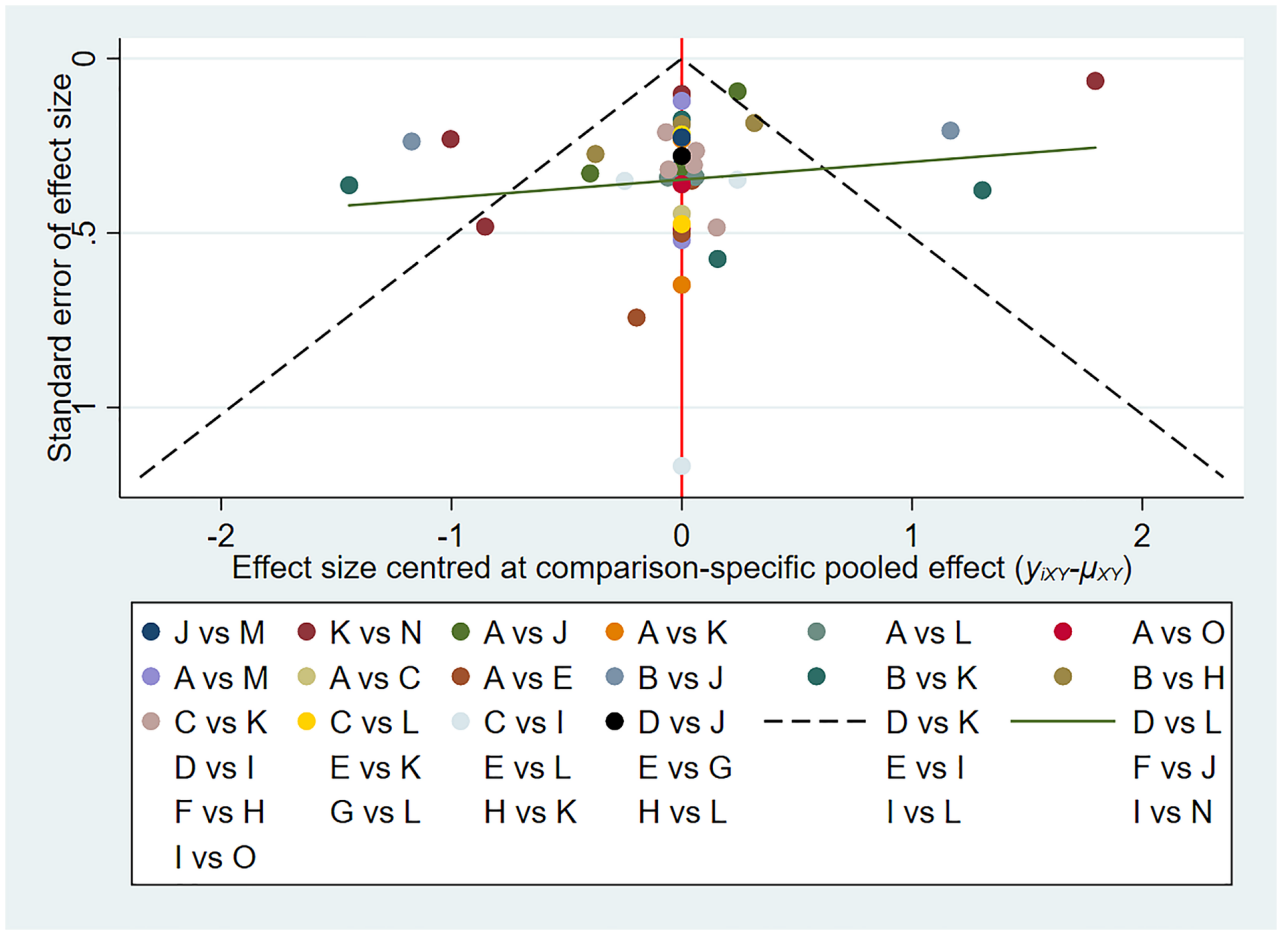
VAS funnel plot of network meta-analysis of acupuncture treatment for post-stroke shoulder-hand syndrome (each dot represents the study we included, and the horizontal axis represents the effect size).

There appears to be no significant publication bias, as shown in the funnel plot (shown in Figure 5).

### 4.2 Fugl–Meyer

#### 4.2.1 Correlation between intervention methods

**Figure 6:**
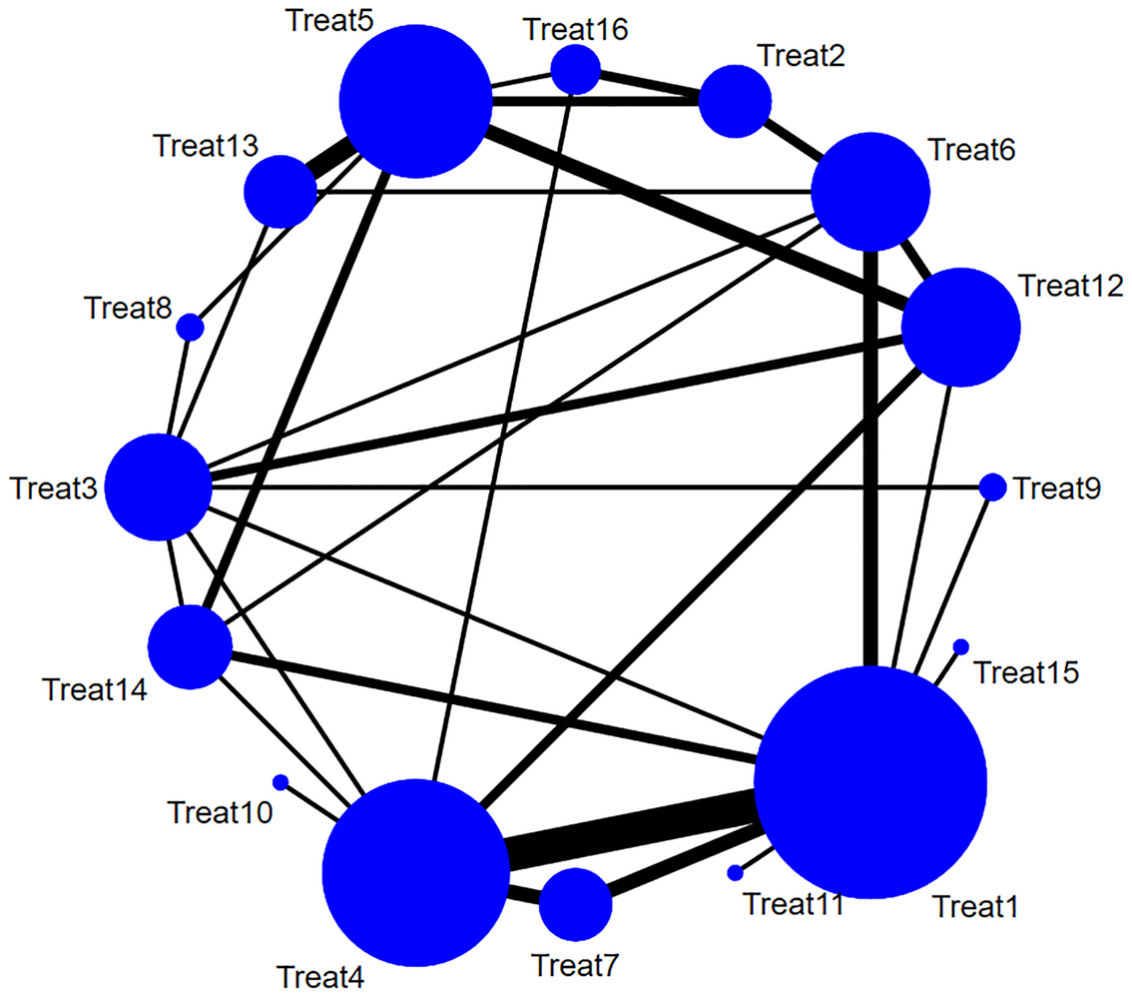
Network diagram of diversified acupuncture treatment plans for the improvement of Fugl–Meyer in post-stroke shoulder-hand syndrome (the larger the dot in the figure, the more samples it contains; the thicker the line between two dots, the more documents are compared between the two groups).

A total of 48 studies (18–32, 34–41, 43–47, 49–67) reported the effect of multiple acupuncture methods on the upper limb motor function of the Fugl–Meyer score in SHS after stroke. The main intervention methods consisted of Treat 1 (rehabilitation), Treat 4 (rehabilitation + acupuncture), Treat 5 (western medicine + rehabilitation + acupuncture), Treat 6 (electroacupuncture), Treat 12 (special needle operation and technique). With the application and development of acupuncture in recent years, some special acupuncture therapies have gradually emerged and have been used in the recovery of upper limb motor function in SHS after stroke, such as Treat 8 (fire needling), Treat 9 (Jin’s three-needle technique), Treat 10 (floating needle), Treat 11 (eye acupuncture), and Treat 15 (western medicine + acupuncture + Chinese medicine). A detailed comparison of the intervention methods is presented in Figure 6.

#### 4.2.2 Synthesized results

**Figure 7:**
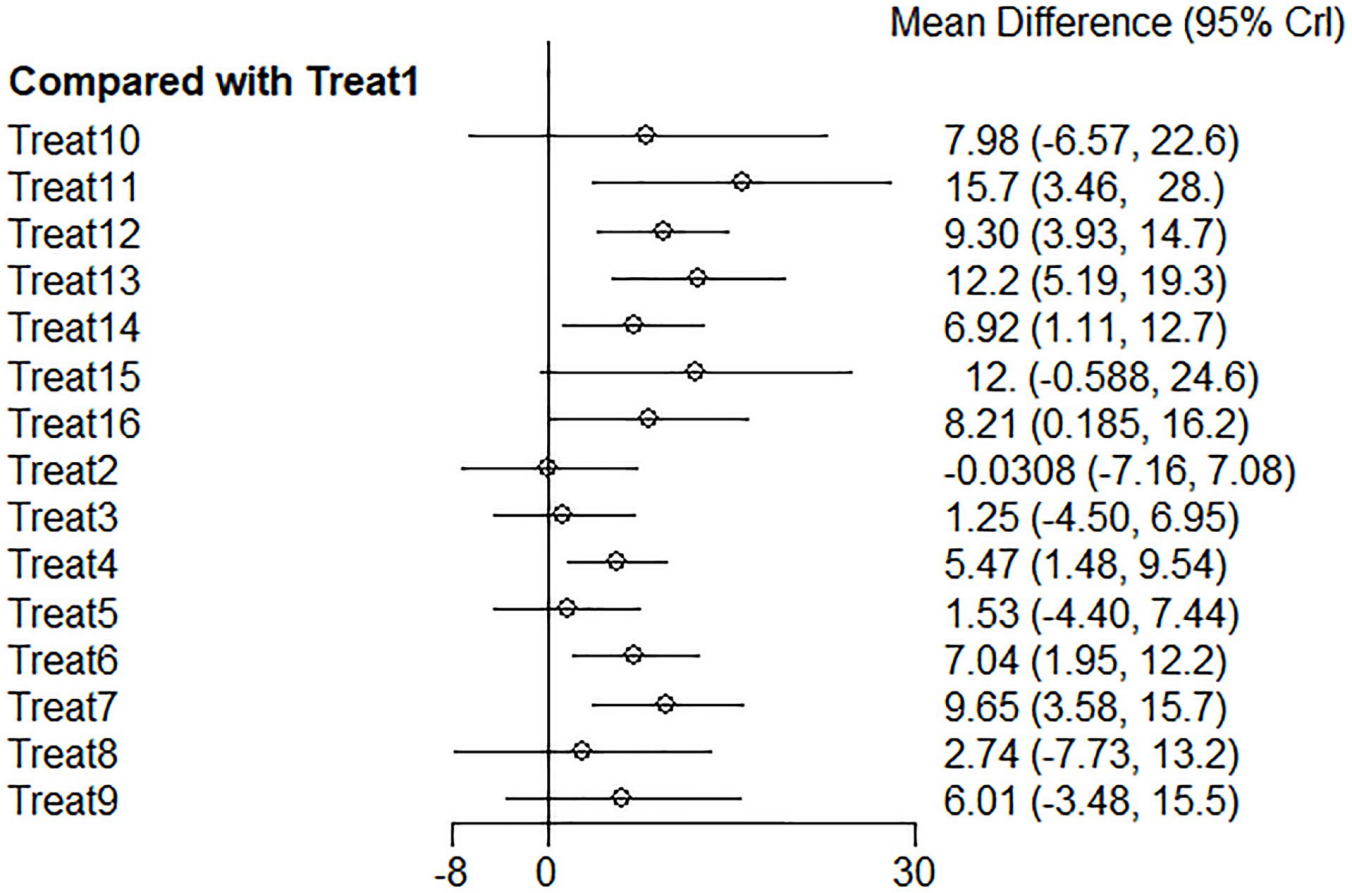
Fugl–Meyer forest plot of network meta-analysis of acupuncture treatments versus rehabilitation therapy for post-stroke shoulder-hand syndrome [the left side of the picture represents the results obtained by comparing various intervention methods with the conventional group Treat 1 (rehabilitation). The horizontal line corresponding to each intervention method represents the confidence interval of the estimated effect. If the confidence interval does not contain a null line, the difference is statistically significant].

The included studies met the overall consistency hypothesis test (*p* > 0.05), so we used a consistency model for Bayesian network meta-analysis. The results of the analysis show that compared to Treat 1 (rehabilitation), Treat 11 (eye acupuncture), Treat 13 (other multi-needle acupuncture combinations), Treat 15 (western medicine + acupuncture + Chinese medicine), Treat 7 (warm acupuncture), Treat 12 (special needle operation and technique), Treat 16 (rehabilitation + catgut embedding), Treat 10 (floating needle), Treat 6 (electroacupuncture), Treat 14 (acupuncture on the non-affected limb), Treat 9 (Jin’s three-needle), Treat 4 (rehabilitation + acupuncture), Treat 8 (fire needling), Treat 5 (western medicine + rehabilitation + acupuncture), and Treat 3 (routine acupuncture) (Figure 7) could significantly increase the Fugl–Meyer score (*p* < 0.05).

Among these effective intervention methods, the top three are Treat 11 (eye acupuncture), Treat 13 (other multi-needle acupuncture combinations), and Treat 15 (western medicine + acupuncture + Chinese medicine), with ranking probabilities of 0.88, 0.84, and 0.75, respectively. At the same time, compared to routine rehabilitation, their improvements in Fugl–Meyer scores were Treat 11 (eye needle) 15.73 (95% CI: 3.4627.95), Treat 13 (other multi-needle acupuncture combinations) 12.22 (95% CI: 5.1919.34), and Treat 15 (western medicine + acupuncture + Chinese medicine) 11.96 (95% CI: −0.59 to 24.63). There was a significant difference between the first two, while Treat 11 (eye needle) showed good therapeutic effects. However, due to the small sample size, this result should be interpreted cautiously. The degree of improvement of the intervention method Treat 15 (western medicine + acupuncture + Chinese medicine) was not significant. The detailed results of pairwise comparisons are provided in Table 3.

**Table 3:** VAS league table of network meta-analysis of acupuncture treatment for post-stroke SHS.

Treat 1	7.98 (−6.57, 22.63)	15.73 (3.46, 27.95)	9.3 (3.93, 14.66)	12.22 (5.19, 19.34)	6.92 (1.11, 12.71)	11.96 (−0.59, 24.63)	8.21 (0.19, 16.17)	−0.03 (−7.16, 7.08)	1.25 (−4.5, 6.95)	5.47 (1.48, 9.54)	1.53 (−4.4, 7.44)	7.04 (1.95, 12.16)	9.65 (3.58, 15.74)	2.74 (−7.73, 13.24)	6.01 (−3.48, 15.55)
−7.98 (−22.63, 6.57)	Treat 10	7.69 (−11.37, 26.71)	1.29 (−13.84, 16.34)	4.24 (−11.61, 20.05)	−1.06 (−16.51, 14.27)	3.99 (−15.27, 23.32)	0.21 (−15.93, 16.22)	−8 (−23.9, 7.8)	−6.76 (−22.12, 8.48)	−2.5 (−16.6, 11.46)	−6.47 (−21.82, 8.81)	−0.94 (−16.16, 14.2)	1.64 (−13.69, 16.92)	−5.26 (−22.95, 12.43)	−1.98 (−19.29, 15.11)
−15.73 (−27.95, −3.46)	−7.69 (−26.71, 11.37)	Treat 11	−6.44 (−19.85, 6.96)	−3.51 (−17.59, 10.72)	−8.81 (−22.36, 4.77)	−3.75 (−21.35, 13.82)	−7.53 (−22.17, 7.12)	−15.75 (−29.93, −1.59)	−14.49 (−28.02, −0.96)	−10.25 (−23.12, 2.69)	−14.2 (−27.83, −0.59)	−8.69 (−21.94, 4.63)	−6.09 (−19.78, 7.61)	−12.99 (−29.15, 3.19)	−9.72 (−25.16, 5.87)
−9.3 (−14.66, −3.93)	−1.29 (−16.34, 13.84)	6.44 (−6.96, 19.85)	Treat 12	2.93 (−3.68, 9.63)	−2.37 (−8.76, 4.01)	2.68 (−10.97, 16.39)	−1.09 (−9.09, 6.88)	−9.32 (−16.38, −2.36)	−8.05 (−13.6, −2.53)	−3.81 (−9.33, 1.71)	−7.77 (−12.93, −2.6)	−2.25 (−7.48, 3.02)	0.36 (−7.26, 8.01)	−6.55 (−16.79, 3.73)	−3.28 (−13.4, 6.87)
−12.22 (−19.34, −5.19)	−4.24 (−20.05, 11.61)	3.51 (−10.72, 17.59)	−2.93 (−9.63, 3.68)	Treat 13	−5.29 (−12.79, 2.05)	−0.25 (−14.75, 14.15)	−4.01 (−12.68, 4.52)	−12.25 (−19.98, −4.68)	−10.98 (−17.86, −4.2)	−6.72 (−14.09, 0.52)	−10.69 (−16.05, −5.47)	−5.19 (−11.82, 1.36)	−2.59 (−11.65, 6.38)	−9.47 (−20.24, 1.22)	−6.22 (−17.19, 4.71)
−6.92 (−12.71, −1.11)	1.06 (−14.27, 16.51)	8.81 (−4.77, 22.36)	2.37 (−4.01, 8.76)	5.29 (−2.05, 12.79)	Treat 14	5.04 (−8.79, 18.94)	1.28 (−7.28, 9.86)	−6.95 (−14.67, 0.74)	−5.68 (−12.18, 0.83)	−1.44 (−7.62, 4.76)	−5.4 (−11.49, 0.73)	0.12 (−6, 6.28)	2.72 (−5.29, 10.78)	−4.19 (−14.91, 6.63)	−0.92 (−11.38, 9.64)
−11.96 (−24.63, 0.59)	−3.99 (−23.32, 15.27)	3.75 (−13.82, 21.35)	−2.68 (−16.39, 10.97)	0.25 (−14.15, 14.75)	−5.04 (−18.94, 8.79)	Treat 15	−3.77 (−18.76, 11.11)	−11.99 (−26.52, 2.38)	−10.73 (−24.56, 3.06)	−6.49 (−19.73, 6.65)	−10.44 (−24.35, 3.42)	−4.92 (−18.53, 8.6)	−2.31 (−16.35, 11.59)	−9.23 (−25.57, 7.14)	−5.97 (−21.79, 9.86)
−8.21 (−16.17, −0.19)	−0.21 (−16.22, 15.93)	7.53 (−7.12, 22.17)	1.09 (−6.88, 9.09)	4.01 (−4.52, 12.68)	−1.28 (−9.86, 7.28)	3.77 (−11.11, 18.76)	Treat 16	−8.22 (−15.09, −1.39)	−6.95 (−15.45, 1.53)	−2.72 (−10.59, 5.16)	−6.68 (−13.96, 0.59)	−1.15 (−8.96, 6.65)	1.44 (−8.1, 11.03)	−5.46 (−17.3, 6.42)	−2.18 (−14.03, 9.65)
0.03 (−7.08, 7.16)	8 (−7.8, 23.9)	15.75 (1.59, 29.93)	9.32 (2.36, 16.38)	12.25 (4.68, 19.98)	6.95 (−0.74, 14.67)	11.99 (−2.38, 26.52)	8.22 (1.39, 15.09)	Treat 2	1.27 (−6.3, 8.9)	5.51 (−1.82, 12.85)	1.55 (−4.64, 7.79)	7.07 (0.78, 13.41)	9.68 (0.69, 18.72)	2.77 (−8.38, 14.01)	6.05 (−5.21, 17.34)
−1.25 (−6.95, 4.5)	6.76 (−8.48, 22.12)	14.49 (0.96, 28.02)	8.05 (2.53, 13.6)	10.98 (4.2, 17.86)	5.68 (−0.83, 12.18)	10.73 (−3.06, 24.56)	6.95 (−1.53, 15.45)	−1.27 (−8.9, 6.3)	Treat 3	4.23 (−1.76, 10.33)	0.28 (−5.72, 6.32)	5.79 (−0.05, 11.72)	8.4 (0.48, 16.38)	1.5 (−8.04, 11.08)	4.77 (−4.54, 14.18)
−5.47 (−9.54, −1.48)	2.5 (−11.46, 16.6)	10.25 (−2.69, 23.12)	3.81 (−1.71, 9.33)	6.72 (−0.52, 14.09)	1.44 (−4.76, 7.62)	6.49 (−6.65, 19.73)	2.72 (−5.16, 10.59)	−5.51 (−12.85, 1.82)	−4.23 (−10.33, 1.76)	Treat 4	−3.96 (−10.08, 2.17)	1.55 (−4.17, 7.27)	4.16 (−1.94, 10.28)	−2.75 (−13.37, 7.95)	0.52 (−9.48, 10.53)
−1.53 (−7.44, 4.4)	6.47 (−8.81, 21.82)	14.2 (0.59, 27.83)	7.77 (2.6, 12.93)	10.69 (5.47, 16.05)	5.4 (−0.73, 11.49)	10.44 (−3.42, 24.35)	6.68 (−0.59, 13.96)	−1.55 (−7.79, 4.64)	−0.28 (−6.32, 5.72)	3.96 (−2.17, 10.08)	Treat 5	5.52 (−0.07, 11.09)	8.11 (0.07, 16.23)	1.2 (−8.63, 11.11)	4.47 (−5.87, 14.9)
−7.04 (−12.16, −1.95)	0.94 (−14.2, 16.16)	8.69 (−4.63, 21.94)	2.25 (−3.02, 7.48)	5.19 (−1.36, 11.82)	−0.12 (−6.28, 6)	4.92 (−8.6, 18.53)	1.15 (−6.65, 8.96)	−7.07 (−13.41, −0.78)	−5.79 (−11.72, 0.05)	−1.55 (−7.27, 4.17)	−5.52 (−11.09, 0.07)	Treat 6	2.62 (−5.02, 10.2)	−4.3 (−14.74, 6.18)	−1.03 (−11.22, 9.12)
−9.65 (−15.74, −3.58)	−1.64 (−16.92, 13.69)	6.09 (−7.61, 19.78)	−0.36 (−8.01, 7.26)	2.59 (−6.38, 11.65)	−2.72 (−10.78, 5.29)	2.31 (−11.59, 16.35)	−1.44 (−11.03, 8.1)	−9.68 (−18.72, −0.69)	−8.4 (−16.38, −0.48)	−4.16 (−10.28, 1.94)	−8.11 (−16.23, −0.07)	−2.62 (−10.2, 5.02)	Treat 7	−6.89 (−18.79, 4.91)	−3.62 (−14.81, 7.53)
−2.74 (−13.24, 7.73)	5.26 (−12.43, 22.95)	12.99 (−3.19, 29.15)	6.55 (−3.73, 16.79)	9.47 (−1.22, 20.24)	4.19 (−6.63, 14.91)	9.23 (−7.14, 25.57)	5.46 (−6.42, 17.3)	−2.77 (−14.01, 8.38)	−1.5 (−11.08, 8.04)	2.75 (−7.95, 13.37)	−1.2 (−11.11, 8.63)	4.3 (−6.18, 14.74)	6.89 (−4.91, 18.79)	Treat 8	3.27 (−9.87, 16.41)
−6.01 (−15.55, 3.48)	1.98 (−15.11, 19.29)	9.72 (−5.87, 25.16)	3.28 (−6.87, 13.4)	6.22 (−4.71, 17.19)	0.92 (−9.64, 11.38)	5.97 (−9.86, 21.79)	2.18 (−9.65, 14.03)	−6.05 (−17.34, 5.21)	−4.77 (−14.18, 4.54)	−0.52 (−10.53, 9.48)	−4.47 (−14.9, 5.87)	1.03 (−9.12, 11.22)	3.62 (−7.53, 14.81)	−3.27 (−16.41, 9.87)	Treat 9

Treat 1 (rehabilitation), Treat 2 (rehabilitation + western medicine), Treat 3 (routine acupuncture), Treat 4 (rehabilitation + acupuncture), Treat 5 (western medicine + rehabilitation + acupuncture), Treat 6 (electroacupuncture), Treat 7 (warm acupuncture), Treat 8 (fire needling), Treat 9 (Jin’s three-needle technique), Treat 10 (floating needle), Treat 11 (eye acupuncture), Treat 12 (special needle operation and technique), Treat 13 (other multi-needle acupuncture combinations), Treat 14 (acupuncture on the non-affected limb), Treat 15 (western medicine + acupuncture + Chinese medicine), Treat 16 (rehabilitation + catgut embedding), Treat 17 (western medicine + rehabilitation + Chinese medicine), Treat 18 (gangliolysis).

#### 4.2.3 Reporting biases

**Figure 8:**
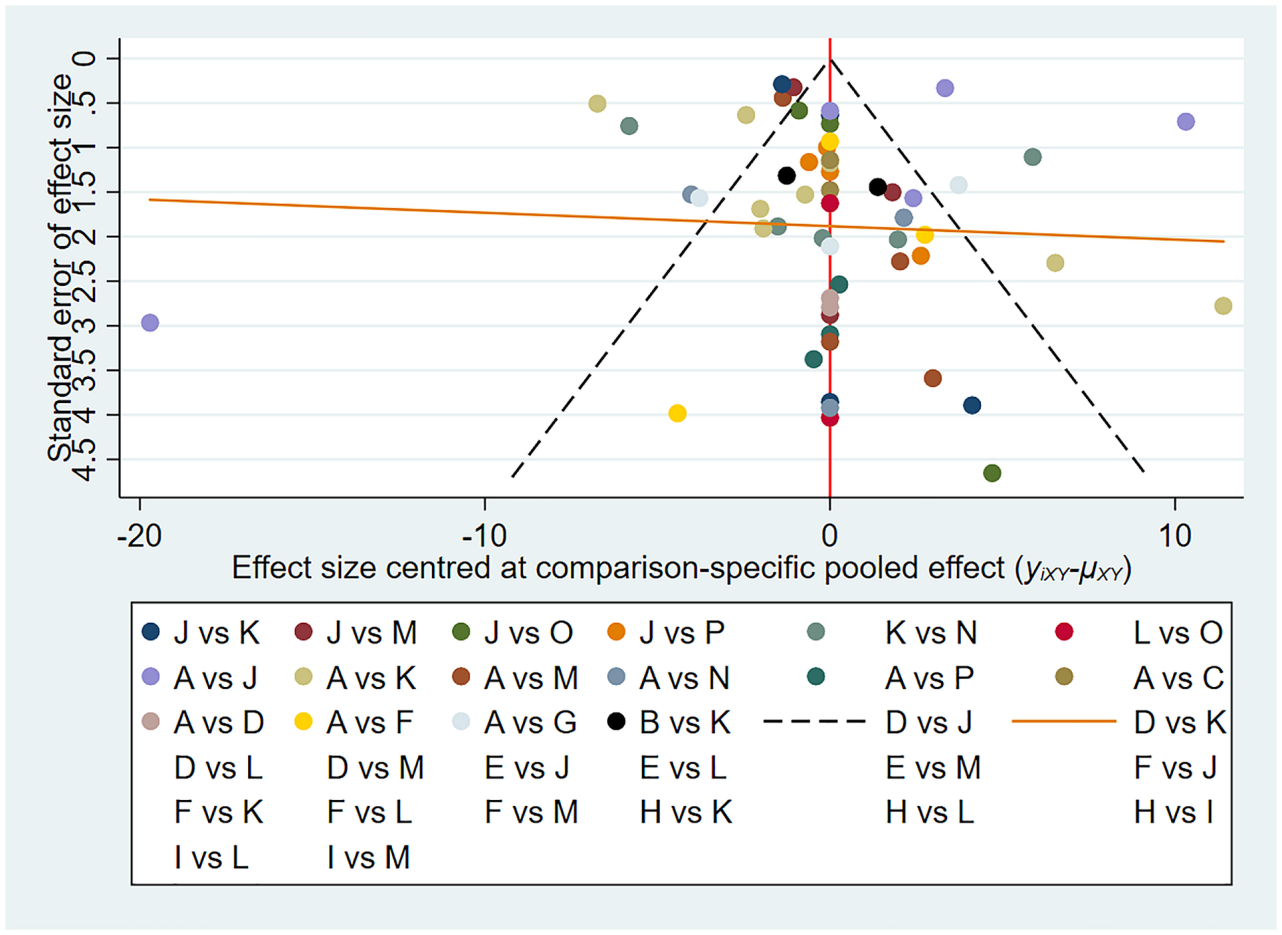
Fugl–Meyer funnel plot of network meta-analysis of acupuncture treatment for post-stroke shoulder-hand syndrome (each dot represents the study we included, and the horizontal axis represents the effect size).

There was no significant publication bias, as shown in the funnel plot (shown in Figure 8).

### 4.3 Secondary outcome measures

We also analyzed the secondary outcome measures of Barthel index and SHSS. Compared to conventional treatment, various interventions did not significantly improve Barthel index or SHSS. The analysis results of secondary outcome measures are presented in Appendix 1.

## 5 Discussion

### 5.1 Summary of the main findings

Our network meta-analysis found that the top five interventions for improving pain according to the VAS scale are floating needle, rehabilitation + catgut embedding, other multi-needle acupuncture combinations, special needle operation and technique, and acupuncture on non-affected limb, which have a significant effect on pain relief. The best five interventions for the Fugl–Meyer score are eye needle, other multi-needle acupuncture combinations, western medicine + acupuncture + traditional Chinese medicine, warming needles, and special needle operation and technique, which could significantly improve upper limb motor function.

We found that rehabilitation + catgut embedding was effective in pain relief. Previous studies have also reviewed this intervention, and the analgesic principle may be a continuous stimulation by absorbable surgical sutures at acupoints, leading to a sterile inflammatory response, and ultimately promoting tissue regeneration (66, 68).

### 5.2 Comparison with previous studies (other reviews)

Although previous studies have also attempted to analyze the effects of acupuncture treatment on post-stroke SHS, some of them only explored the relative effectiveness of a single type of acupuncture or a certain acupuncture combination with rehabilitation and the control group (69–72). Post-stroke SHS is caused by multiple etiologies. Single therapy may not achieve the ideal pain relief. The clinical practice tends to combine multiple intervention methods, which can help explore more reasonable and effective treatment options. The number of types of acupuncture and studies included in existing network meta-analyses is limited (73), but there are many types of acupuncture. Furthermore, the above studies did not consider the existing types of acupuncture or combined acupuncture treatment methods to comprehensively compare various acupuncture interventions.

The mainstream treatment for post-stroke SHS is still rehabilitation therapy. As an indispensable intervention for treating this disease, it focuses on function recovery. Meanwhile, the obstacles affecting function recovery should be tackled to reduce the chances of patients refusing rehabilitation. Aerobic exercise of the upper limbs is found to reduce pain during the day and during exercise (74, 75). Some studies have reported that the combination of two or more therapies may be more effective than rehabilitation alone. Acupuncture combined with rehabilitation training can significantly help in reducing pain scores, promoting limb motor function recovery, and improving overall effectiveness (69–71, 76).

Drug therapy includes oral calcium channel modulators, anti-inflammatory, analgesic, antidepressants, or invasive injections of corticosteroids, bisphosphonates, and botulinum toxin (77, 78). Although drug treatment is convenient and quick, it has limited effects, and long-term use can produce adverse reactions such as hepatorenal and cardiac toxicity, excessive sedation, and drug resistance (79). Some drugs also have the possibility of recurrence after withdrawal and cannot fundamentally control and treat this disease (78, 80–83). In contrast, acupuncture has the advantages of high safety and no toxic side effects and is more easily accepted by patients with heart, liver, and kidney damage.

Neural stimulation, nerve block, and ganglion radiofrequency ablation can avoid the adverse systemic effects induced by drug treatment. Nonetheless, invasive surgery has certain adverse reactions and risks, and the implanted stimulation device may also have problems such as wire disconnection, requiring regular battery replacement (78, 84). A study has even found that the effects of standard high-frequency neurostimulation may be worse than acupuncture or other types of electrical stimulation, and acupuncture can become a promising treatment method for patients with drug-resistant neuropathic pain (84). The nerve block needs to be carried out under ultrasound guidance to improve safety. After a single treatment session, 61.8% of patients still have pain recurrence within 3 months. For chronic pain, it cannot achieve fundamental relief (78, 85–87). Ganglion radiofrequency ablation may cause risks of sensory and motor dysfunction and nerve damage, with small clinical improvement in the VAS score of patients with chronic root pain (88). Its long-term therapeutic effects are still controversial.

The treatment of post-stroke SHS is to relieve pain in the early stage of the disease without affecting the motor function and rehabilitation process of the affected limb. Conventional analgesic drugs, such as opioids and anti-inflammatory drugs, have a variety of side effects such as reduced motor function, induced hyperalgesia, and lethargy (89). At present, acupuncture is widely used for stroke rehabilitation and various pain-related diseases in China, the United States, Europe, and North America. Although acupuncture is an invasive treatment, the incidence of acupuncture-induced adverse reactions is significantly lower than that of many drugs or other recognized medical interventions according to the consensus report for the NIH Conference (90). Acupuncture can reduce the excitability of the pain pathway by reducing excitatory neurotransmitters and promoting inhibitory neurotransmitters (91, 92). Electroacupuncture has been shown to induce peripheral opioid receptors in rats, producing long-term analgesic effects for up to 144 h, improving motor function, and normalizing neurotransmitter metabolism (93). A preclinical study has also shown that acupuncture’s antihyperalgesic effects are associated with 10 peripheral receptors and 8 neurotransmitters (94).

### 5.3 Advantages and limitations of the study

This systematic review is the first to focus on the advantages of different acupuncture treatment plans to broaden the selection of feasible treatment options in clinical practice. The advantages of different acupuncture treatments are different. There are currently no studies comparing the effectiveness of various types of acupuncture for post-stroke SHS directly or indirectly. Our network meta-analysis includes a large number of studies, integrating various acupuncture intervention methods and comparing the improvement of different acupuncture therapies for post-stroke SHS based on VAS, Fugl–Meyer, Barthel, and SHSS scales. The aim is to provide more comprehensive clinical practice references for acupuncture treatment for post-stroke SHS.

However, there are also several limitations. Since acupuncture is a traditional Chinese treatment method, its promotion in the world is still limited to some extent. The included studies are mainly from China and lack ethnic diversity. Therefore, research and exploration outside China are required to enhance clinical promotion and applicability. The multi-center studies included in our research are relatively few. To acquire more convincing evidence, future studies are desired to verify these results.

## 6 Conclusion

From the results, we found that the combination of acupuncture and rehabilitation is more effective in improving the overall effectiveness of post-stroke SHS than simple rehabilitation intervention, especially in relieving pain and improving motor function. Floating needle acupuncture and catgut embedding + rehabilitation show good performance in pain relief, but the sample size of floating needle acupuncture is small. Large sample sizes and high-quality studies are required for verification in the future. Most notably, other multi-needle acupuncture combinations and special needle operations and techniques all demonstrate good effects in pain relief and upper limb motor function recovery. This could be related to the inclusion of special needle operation and technique and the combination with the patient’s limb activity during the acupuncture treatment. For patients who fear rehabilitation exercise due to pain, these two acupuncture methods can be considered as the first options. Western medicine + acupuncture + traditional Chinese medicine is also effective in improving Fugl–Meyer, Barthel, and SHSS scores. However, since it is not reflected in the VAS scale, there is currently a lack of effective exploration of its pain relief effect. It is recommended that future research could supplement this gap.

In conclusion, multiple acupuncture treatments have significant effects in treating pain and improving upper limb motor function after a stroke, with fewer adverse events. Hence, acupuncture can be promoted as a treatment method. More high-quality, multi-center collaborative, large-sample studies are warranted to verify our results in future.

## Data availability statement

The original contributions presented in the study are included in the article/Supplementary material, further inquiries can be directed to the corresponding author.

## Author contributions

TH: Conceptualization, Data curation, Formal analysis, Investigation, Methodology, Project administration, Software, Validation, Visualization, Writing – original draft. HY: Data curation, Funding acquisition, Investigation, Project administration, Supervision, Writing – review & editing. JH: Formal analysis, Investigation, Supervision, Writing – review & editing. NW: Conceptualization, Data curation, Formal analysis, Resources, Writing – review & editing. CZ: Data curation, Formal analysis, Investigation, Writing – review & editing. XH: Data curation, Investigation, Writing – review & editing. XT: Data curation, Investigation, Writing – review & editing. YaL: Data curation, Investigation, Writing – review & editing. YJ: Data curation, Investigation, Project administration, Writing – review & editing. XW: Data curation, Investigation, Project administration, Writing – review & editing. YY: Resources, Supervision, Writing – review & editing. YiL: Funding acquisition, Project administration, Resources, Supervision, Writing – review & editing. SY: Data curation, Investigation, Writing – review & editing. YM: Data curation, Investigation, Writing – review & editing. SL: Data curation, Investigation, Writing – review & editing. JZ: Data curation, Investigation, Writing – review & editing. YH: Conceptualization, Funding acquisition, Resources, Supervision, Validation, Writing – review & editing.
