# Supplementary material for: Effectiveness of acupuncture for pain relief in shoulder-hand syndrome after stroke: a systematic evaluation and Bayesian network meta-analysis
Source: Front Neurol. 2023 Nov 17;14:1268626. doi: 10.3389/fneur.2023.1268626 (PMC10693460; doi:10.3389/fneur.2023.1268626)
Supplement: Supplementary file 1 [file Table_1.DOCX]

**Appendix1** Secondary outcome indicators

1. Barthel Index (BI)

(1) Correlation among intervention methods

Sixteen studies reported the improvement effect of multiple acupuncture methods on BI in SHS (shoulder-hand syndrome) after stroke. The main intervention methods consisted of Treat 1 (rehabilitation), Treat 4 (rehabilitation + acupuncture) and Treat 5 (western medicine + rehabilitation + acupuncture). With the promotion and application of acupuncture in post-stroke rehabilitation and recovery, Treat 3 (routine acupuncture), Treat 6 (electroacupuncture), Treat 7 (warm acupuncture) and Treat 11 (eye acupuncture) have gradually emerged and have been used in the recovery of self-care ability in the daily life of patients with SHS after stroke. The detailed intervention methods are provided in Figure A.


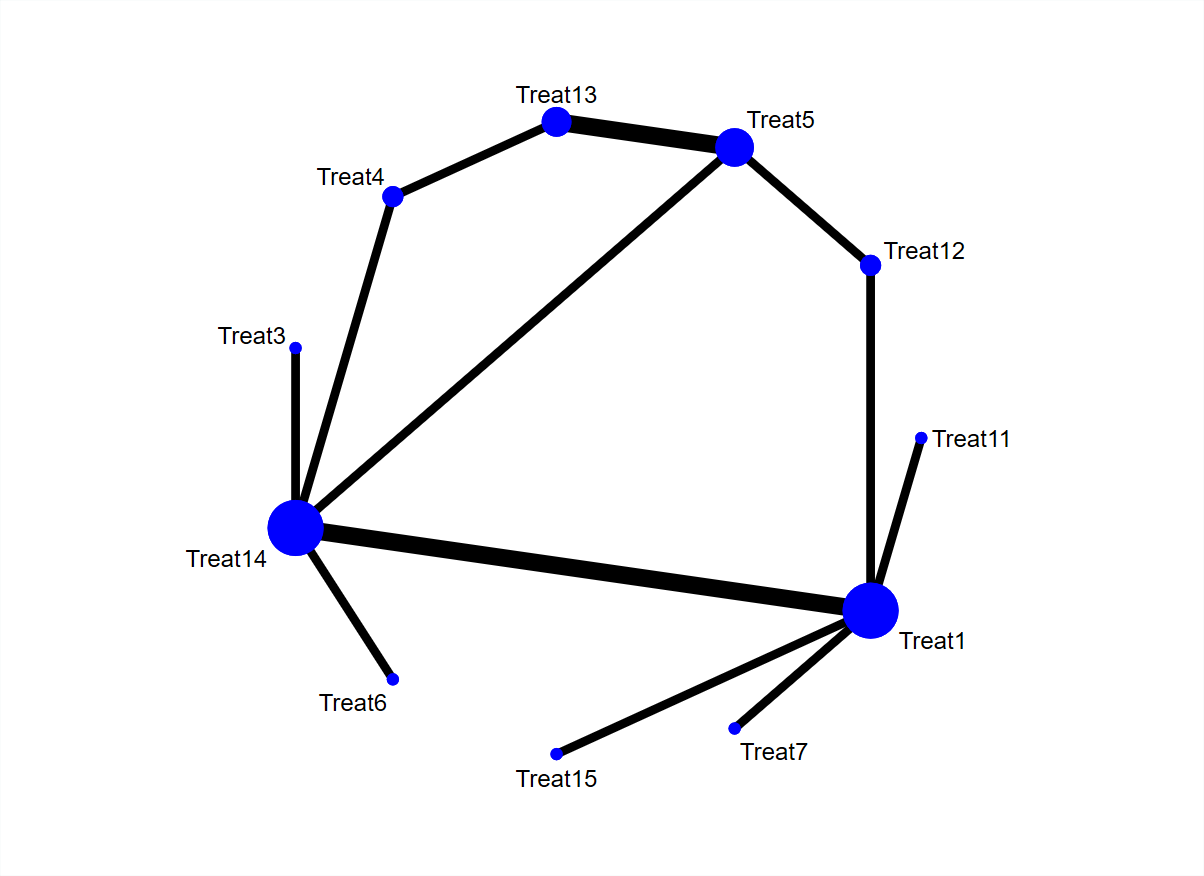


Figure A: network diagram of diversified acupuncture treatment plans for the improvement of BI in post-stroke shoulder-hand syndrome (Note: The larger the dot in the figure, the more samples it contains; The thicker the line between two dots, the more documents are compared between the two groups.)

(2) Synthesized results

The included studies met the overall consistency hypothesis test (P>0.05), so we used a consistency model for Bayesian network meta-analysis. The results of the analysis show that compared to Treat 1 (rehabilitation), Treat 15 (western medicine + acupuncture + Chinese medicine), Treat 13 (other multi-needle acupuncture combination), Treat 14 (acupuncture on non-affected limb), Treat 12 (special needle operation and technique), Treat 11 (eye acupuncture), Treat 6 (electroacupuncture), Treat 7 (warm acupuncture), Treat 4 (rehabilitation + acupuncture), Treat 5 (western medicine + rehabilitation + acupuncture), and Treat 3 (routine acupuncture) (Figure 2) were effective intervention methods that could significantly increase the Barthel score (P < 0.05).

As shown in the league table, there was no significant difference among these effective intervention methods in the improvement of the Barthel scale (Figure B and Table A).


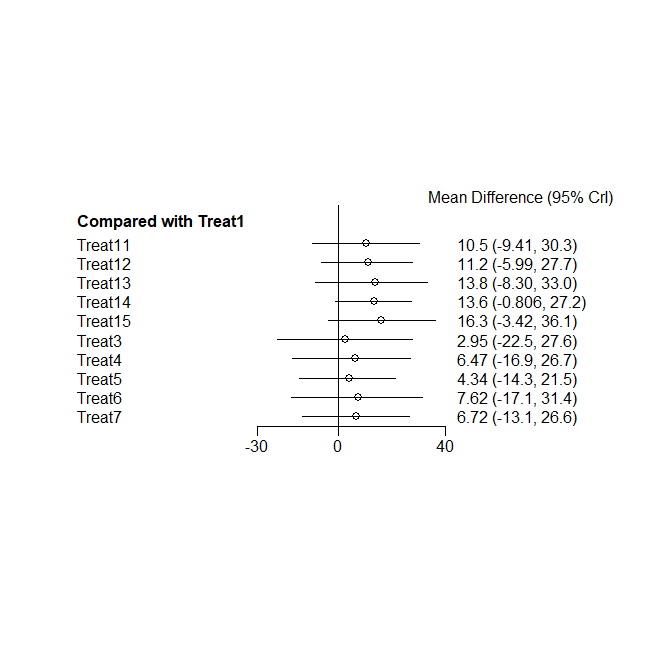


Figure B Forest plot of network meta-analysis of acupuncture treatment versus rehabilitation therapy for post-stroke SHS(Note: The left side of the picture represents the results obtained after comparing various intervention methods with the conventional group Treat 1(rehabilitation). The horizontal line for each intervention represents the confidence interval for estimating the effect size. If the confidence interval does not contain a null line, the difference is statistically significant.)

Table A League table of network meta-analysis of acupuncture treatment for post-stroke SHS

| Treat1 | 10.51 (-9.41, 30.27) | 11.17 (-5.99, 27.7) | 13.78 (-8.3, 33.04) | 13.58 (-0.81, 27.15) | 16.3 (-3.42, 36.07) | 2.95 (-22.47, 27.6) | 6.47 (-16.86, 26.68) | 4.34 (-14.33, 21.47) | 7.62 (-17.05, 31.38) | 6.72 (-13.15, 26.64) |
| --- | --- | --- | --- | --- | --- | --- | --- | --- | --- | --- |
| -10.51 (-30.27, 9.41) | Treat11 | 0.65 (-25.66, 26.54) | 3.31 (-26.84, 30.64) | 3.08 (-21.44, 27.01) | 5.79 (-22.1, 34.08) | -7.58 (-39.88, 24.02) | -3.96 (-35, 24.01) | -6.18 (-33.67, 19.99) | -2.87 (-34.68, 27.87) | -3.74 (-31.92, 24.4) |
| -11.17 (-27.7, 5.99) | -0.65 (-26.54, 25.66) | Treat12 | 2.67 (-19.18, 22.01) | 2.43 (-16.03, 20.66) | 5.13 (-20.48, 31.58) | -8.21 (-36.18, 19.57) | -4.66 (-29, 17.22) | -6.81 (-24.03, 9.6) | -3.53 (-30.74, 23.42) | -4.46 (-30.24, 22.17) |
| -13.78 (-33.04, 8.3) | -3.31 (-30.64, 26.84) | -2.67 (-22.01, 19.18) | Treat13 | -0.2 (-17.16, 18.74) | 2.45 (-24.65, 32.78) | -10.83 (-37.3, 17.75) | -7.24 (-24.85, 9.87) | -9.51 (-21.59, 4.23) | -6.22 (-31.95, 21.62) | -7.12 (-34.32, 23.37) |
| -13.58 (-27.15, 0.81) | -3.08 (-27.01, 21.44) | -2.43 (-20.66, 16.03) | 0.2 (-18.74, 17.16) | Treat14 | 2.69 (-21.1, 27.46) | -10.69 (-31.53, 10.26) | -7.14 (-26.42, 9.88) | -9.26 (-24.88, 5.68) | -5.99 (-25.69, 13.78) | -6.87 (-30.68, 18.06) |
| -16.3 (-36.07, 3.42) | -5.79 (-34.08, 22.1) | -5.13 (-31.58, 20.48) | -2.45 (-32.78, 24.65) | -2.69 (-27.46, 21.1) | Treat15 | -13.35 (-45.79, 18.18) | -9.73 (-41.07, 17.77) | -11.93 (-39.55, 13.93) | -8.7 (-40.56, 22.1) | -9.57 (-37.68, 18.41) |
| -2.95 (-27.6, 22.47) | 7.58 (-24.02, 39.88) | 8.21 (-19.57, 36.18) | 10.83 (-17.75, 37.3) | 10.69 (-10.26, 31.53) | 13.35 (-18.18, 45.79) | Treat3 | 3.52 (-25.27, 30.13) | 1.39 (-24.76, 26.8) | 4.69 (-24.06, 33.6) | 3.78 (-27.78, 36.3) |
| -6.47 (-26.68, 16.86) | 3.96 (-24.01, 35) | 4.66 (-17.22, 29) | 7.24 (-9.87, 24.85) | 7.14 (-9.88, 26.42) | 9.73 (-17.77, 41.07) | -3.52 (-30.13, 25.27) | Treat4 | -2.19 (-20.01, 17.52) | 1.05 (-24.6, 29.14) | 0.18 (-27.67, 31.61) |
| -4.34 (-21.47, 14.33) | 6.18 (-19.99, 33.67) | 6.81 (-9.6, 24.03) | 9.51 (-4.23, 21.59) | 9.26 (-5.68, 24.88) | 11.93 (-13.93, 39.55) | -1.39 (-26.8, 24.76) | 2.19 (-17.52, 20.01) | Treat5 | 3.22 (-21.39, 28.61) | 2.34 (-23.71, 30.12) |
| -7.62 (-31.38, 17.05) | 2.87 (-27.87, 34.68) | 3.53 (-23.42, 30.74) | 6.22 (-21.62, 31.95) | 5.99 (-13.78, 25.69) | 8.7 (-22.1, 40.56) | -4.69 (-33.6, 24.06) | -1.05 (-29.14, 24.6) | -3.22 (-28.61, 21.39) | Treat6 | -0.89 (-31.75, 31.03) |
| -6.72 (-26.64, 13.15) | 3.74 (-24.4, 31.92) | 4.46 (-22.17, 30.24) | 7.12 (-23.37, 34.32) | 6.87 (-18.06, 30.68) | 9.57 (-18.41, 37.68) | -3.78 (-36.3, 27.78) | -0.18 (-31.61, 27.67) | -2.34 (-30.12, 23.71) | 0.89 (-31.03, 31.75) | Treat7 |

Note: Treat 1 (rehabilitation), Treat 2 (rehabilitation + western medicine), Treat 3 (routine acupuncture), Treat 4 (rehabilitation + acupuncture), Treat 5 (western medicine + rehabilitation + acupuncture), Treat 6 (electroacupuncture), Treat 7 (warm acupuncture), Treat 8 (fire needling), Treat 9 (Jin's three-needle technique), Treat 10 (floating needle), Treat 11 (eye acupuncture), Treat 12 (special needle operation and technique), Treat 13 (other multi-needle acupuncture combination), Treat 14 (acupuncture on non-affected limb), Treat 15 (western medicine + acupuncture + Chinese medicine), Treat 16 (rehabilitation + catgut embedding), Treat 17 (western medicine + rehabilitation + Chinese medicine), Treat 18 (gangliolysis)

(3) Reporting biases

There appears to be no significant publication bias, as shown in the funnel plot. The detailed intervention methods are shown in Figure C.


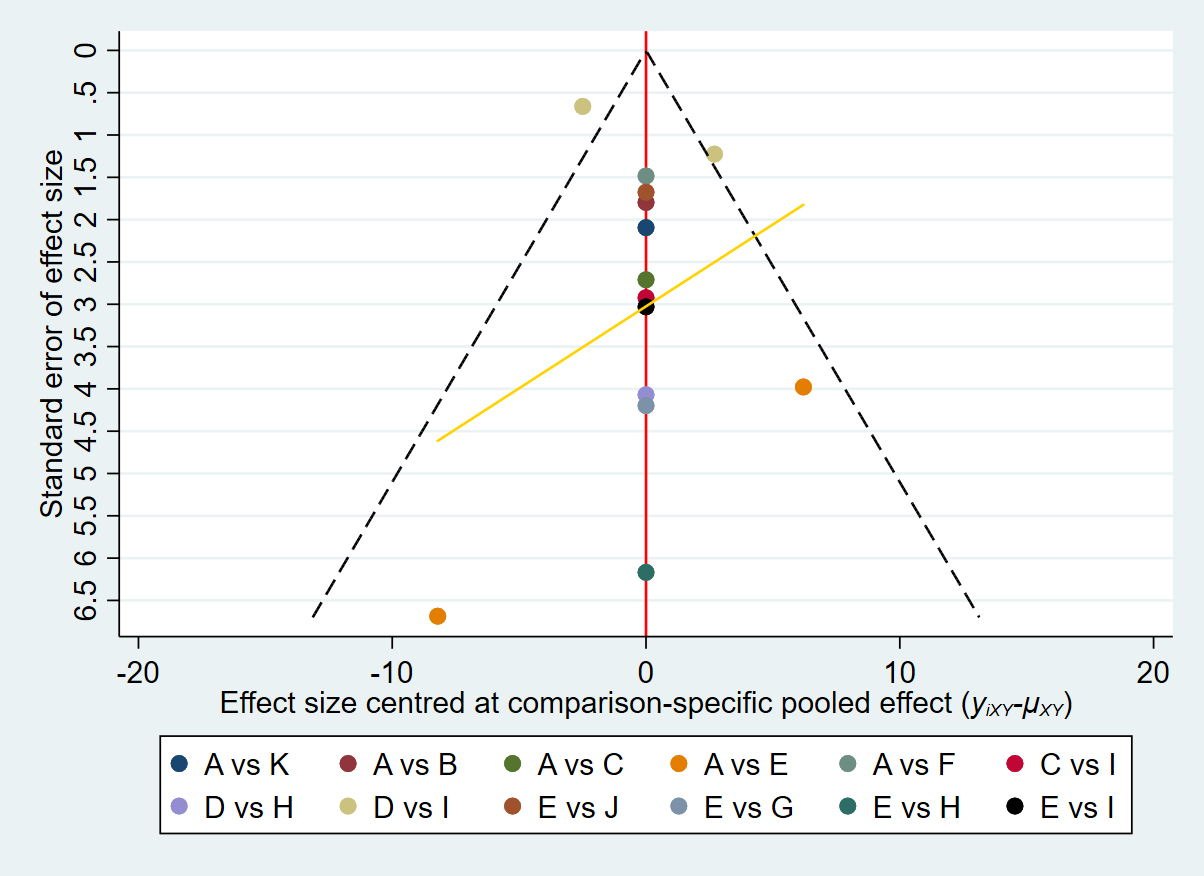


Figure C Funnel plot of network meta-analysis of acupuncture treatment for post-stroke SHS(Note: Each dot represents the study we included, and the horizontal axis represents the effect size.)

2. Shoulder-Hand Syndrome Evaluation Scale (SHSS)

(1) Correlation among intervention methods

Seven included studies have reported the reduction effect of different multiple acupuncture methods on SHSS in post-stroke SHS, among which the most important intervention methods are Treat 1 (rehabilitation), Treat 4 (rehabilitation + acupuncture) and Treat 7 (warm acupuncture). The detailed intervention methods are shown in Figure D.


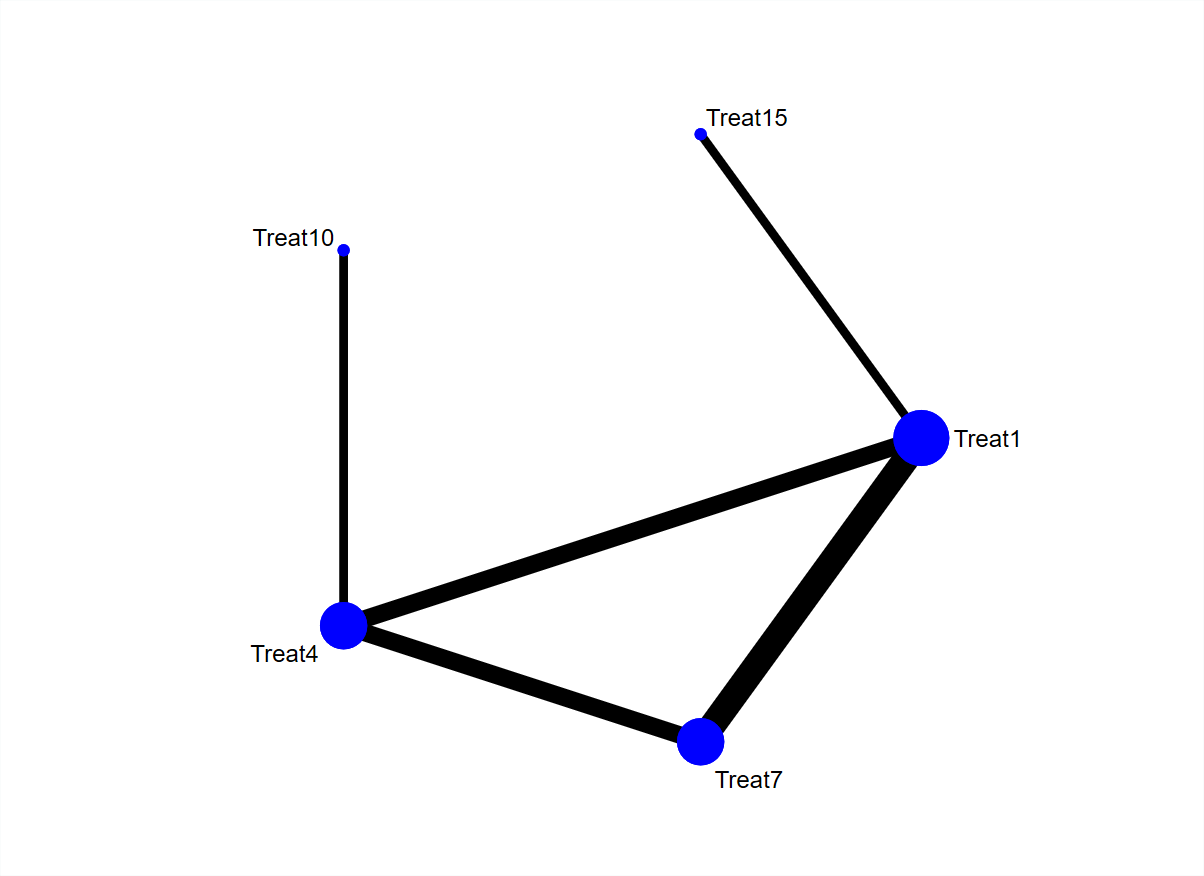


**Figure D**: network relationship diagram of diversified acupuncture treatment plans for SHSS in post-stroke SHS(Note: The larger the dot in the figure, the more samples it contains; The thicker the line between two dots, the more documents are compared between the two groups.

(2) Synthesized results

The included studies met the overall consistency hypothesis test (P > 0.05), so we used a consistency model for Bayesian network meta-analysis. The results of the analysis show that compared to Treat 1 (rehabilitation), among the effective intervention methods of Treat 10 (floating needle), Treat 7 (warm acupuncture), Treat 15 (western medicine + acupuncture + Chinese medicine), and Treat 4 (rehabilitation + acupuncture), Treat 1 (rehabilitation) + Treat 10 (floating needle), Treat 10 (floating needle) + Treat 15 (western medicine + acupuncture + Chinese medicine), Treat 15 (western medicine + acupuncture + Chinese medicine) + Treat 4 (rehabilitation + acupuncture) and Treat 4 (rehabilitation + acupuncture) + Treat 7 (warm acupuncture) (Figure 5) could significantly reduce the SHSS score (P < 0.05).

Among these effective intervention methods, the top three were Treat 10 (floating needle), Treat 7 (warm acupuncture), and Treat 15 (western medicine + acupuncture + Chinese medicine), with ranking probabilities of... At the same time, compared to routine rehabilitation, the improvement in SHSS was: Treat 7 (warm acupuncture) 1.14 (0.27, 1.95). There were significant differences in pairwise comparisons of these effective intervention methods in the league table (Figure E and Table B).


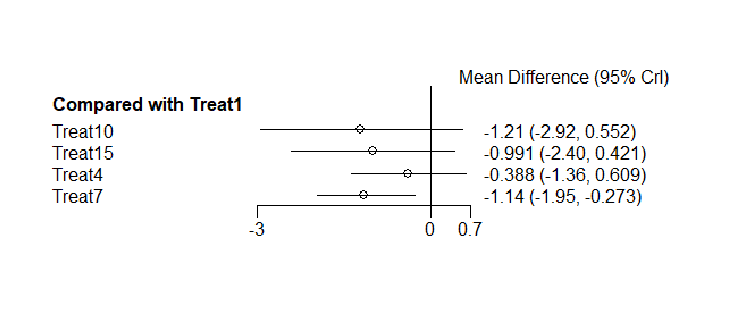


Figure E. Forest plot of network meta-analysis of acupuncture treatment versus rehabilitation therapy for post-stroke SHS(Note: The left side of the picture represents the results obtained by comparing various intervention methods with the conventional group Treat1(rehabilitation). The horizontal line corresponding to each intervention method represents the confidence interval of the estimated effect. If the confidence interval does not contain a null line, the difference is statistically significant).

**Table B.** League table of network meta-analysis of acupuncture treatment for post-stroke SHS

| Treat1 | -1.21 (-2.92, 0.55) | -0.99 (-2.4, 0.42) | -0.39 (-1.36, 0.61) | -1.14 (-1.95, -0.27) |
| --- | --- | --- | --- | --- |
| 1.21 (-0.55, 2.92) | Treat10 | 0.22 (-2.05, 2.45) | 0.82 (-0.62, 2.25) | 0.07 (-1.66, 1.82) |
| 0.99 (-0.42, 2.4) | -0.22 (-2.45, 2.05) | Treat15 | 0.6 (-1.11, 2.34) | -0.15 (-1.76, 1.52) |
| 0.39 (-0.61, 1.36) | -0.82 (-2.25, 0.62) | -0.6 (-2.34, 1.11) | Treat4 | -0.75 (-1.72, 0.25) |
| 1.14 (0.27, 1.95) | -0.07 (-1.82, 1.66) | 0.15 (-1.52, 1.76) | 0.75 (-0.25, 1.72) | Treat7 |

Note: Treat 1 (rehabilitation), Treat 2 (rehabilitation + western medicine), Treat 3 (routine acupuncture), Treat 4 (rehabilitation + acupuncture), Treat 5 (western medicine + rehabilitation + acupuncture), Treat 6 (electroacupuncture), Treat 7 (warm acupuncture), Treat 8 (fire needling), Treat 9 (Jin's three-needle technique), Treat 10 (floating needle), Treat 11 (eye acupuncture), Treat 12 (special needle operation and technique), Treat 13 (other multi-needle acupuncture combination), Treat 14 (acupuncture on non-affected limb), Treat 15 (western medicine + acupuncture + Chinese medicine), Treat 16 (rehabilitation + catgut embedding), Treat 17 (western medicine + rehabilitation + Chinese medicine), Treat 18 (gangliolysis)

(3) Reporting biases

There appears to be no significant publication bias, as shown in the funnel plot. The detailed intervention methods are shown in Figure F.


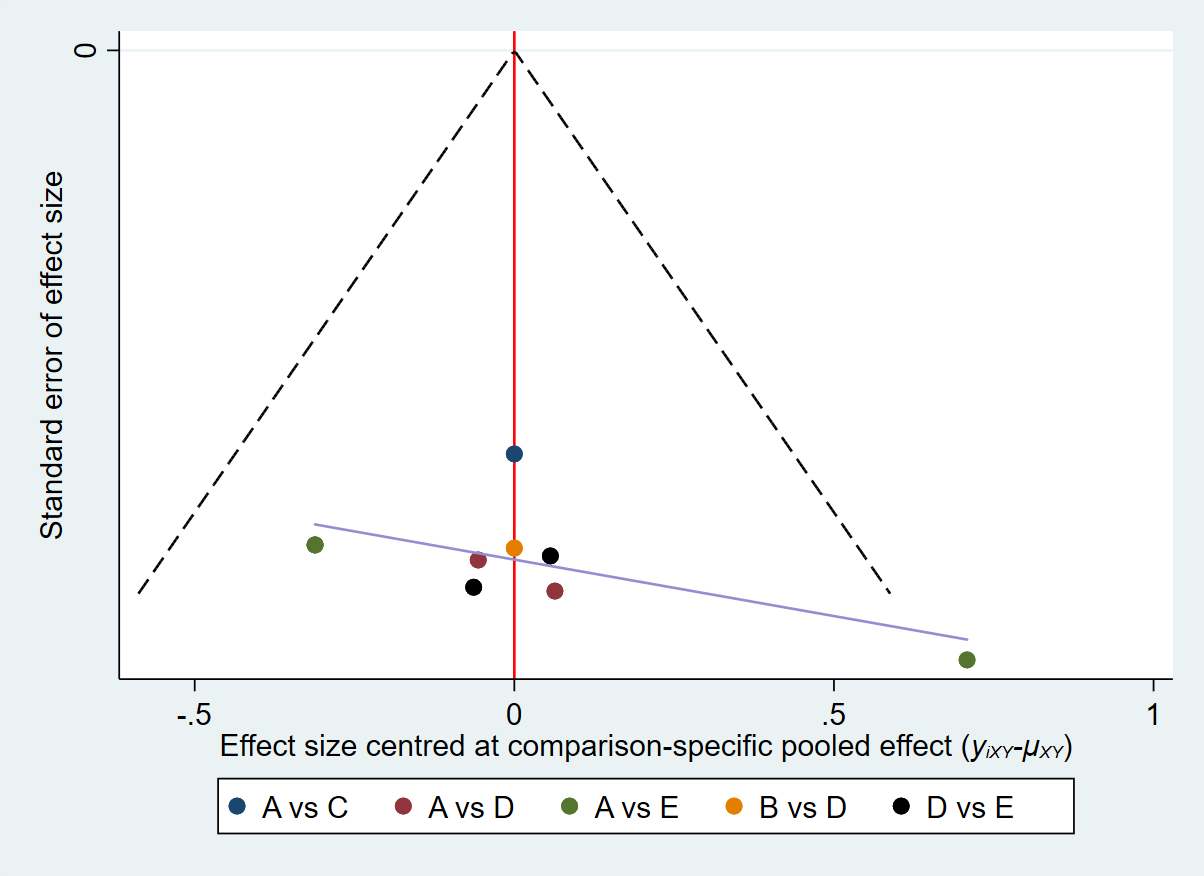


**Figure F.** Funnel plot of network meta-analysis of acupuncture treatment for post-stroke SHS (Note: Each dot represents the study we included, and the horizontal axis represents the effect size.)
