# Supplementary material for: Effectiveness of acupuncture for pain relief in shoulder-hand syndrome after stroke: a systematic evaluation and Bayesian network meta-analysis
Source: Front Neurol. 2023 Nov 17;14:1268626. doi: 10.3389/fneur.2023.1268626 (PMC10693460; doi:10.3389/fneur.2023.1268626)
Supplement: Supplementary file 2 [file Table_2.DOCX]

# Table S1 Literature search strategy

**1.Pubmed**

| Search number | Query | Results |
| --- | --- | --- |
| 1 | "Stroke"[Mesh] | 168,600 |
| 2 | (((((((Stroke[Title/Abstract]) OR (Strokes[Title/Abstract])) OR (Cerebrovascular Accident[Title/Abstract])) OR (Cerebrovascular Accidents[Title/Abstract])) OR (Cerebrovascular Apoplexy[Title/Abstract])) OR (Brain Vascular Accident[Title/Abstract])) OR (Brain Vascular Accidents[Title/Abstract])) OR (Apoplexy[Title/Abstract]) | 319,430 |
| 3 | ("Stroke"[Mesh]) OR ((((((((Stroke[Title/Abstract]) OR (Strokes[Title/Abstract])) OR (Cerebrovascular Accident[Title/Abstract])) OR (Cerebrovascular Accidents[Title/Abstract])) OR (Cerebrovascular Apoplexy[Title/Abstract])) OR (Brain Vascular Accident[Title/Abstract])) OR (Brain Vascular Accidents[Title/Abstract])) OR (Apoplexy[Title/Abstract])) | 356,925 |
| 4 | "Reflex Sympathetic Dystrophy"[Mesh] | 3,635 |
| 5 | (((((((((((((((((((((((Reflex Sympathetic Dystrophy[Title/Abstract]) OR (complex regional pain syndrome type I[Title/Abstract])) OR (Reflex Sympathetic Dystrophies[Title/Abstract])) OR (Sudek Atrophy[Title/Abstract])) OR (Sympathetic Reflex Dystrophia[Title/Abstract])) OR (Sympathetic Reflex Dystrophias[Title/Abstract])) OR (Type I Complex Regional Pain Syndrome[Title/Abstract])) OR (Sudek's Atrophy[Title/Abstract])) OR (Sudek's Atrophies[Title/Abstract])) OR (Sudeks Atrophy[Title/Abstract])) OR (Sudeks Atrophy[Title/Abstract])) OR (CRPS Type Is[Title/Abstract])) OR (Reflex Sympathetic Dystrophy Syndrome[Title/Abstract])) OR (Cervical Sympathetic Dystrophy[Title/Abstract])) OR (Cervical Sympathetic Dystrophies[Title/Abstract])) OR (Shoulder-Hand Syndrome[Title/Abstract])) OR (Shoulder Hand Syndrome[Title/Abstract])) OR (Shoulder-Hand Syndromes[Title/Abstract])) OR (Algodystrophic Syndrome[Title/Abstract])) OR (Algodystrophy[Title/Abstract])) OR (Algodystrophies[Title/Abstract])) OR (shoulder arm syndrome[Title/Abstract])) OR (sympathetic dystrophy syndrome[Title/Abstract])) OR (sympathetic reflex dystrophy[Title/Abstract]) | 115,565 |
| 6 | ("Reflex Sympathetic Dystrophy"[Mesh]) OR ((((((((((((((((((((((((Reflex Sympathetic Dystrophy[Title/Abstract]) OR (complex regional pain syndrome type I[Title/Abstract])) OR (Reflex Sympathetic Dystrophies[Title/Abstract])) OR (Sudek Atrophy[Title/Abstract])) OR (Sympathetic Reflex Dystrophia[Title/Abstract])) OR (Sympathetic Reflex Dystrophias[Title/Abstract])) OR (Type I Complex Regional Pain Syndrome[Title/Abstract])) OR (Sudek's Atrophy[Title/Abstract])) OR (Sudek's Atrophies[Title/Abstract])) OR (Sudeks Atrophy[Title/Abstract])) OR (Sudeks Atrophy[Title/Abstract])) OR (CRPS Type Is[Title/Abstract])) OR (Reflex Sympathetic Dystrophy Syndrome[Title/Abstract])) OR (Cervical Sympathetic Dystrophy[Title/Abstract])) OR (Cervical Sympathetic Dystrophies[Title/Abstract])) OR (Shoulder-Hand Syndrome[Title/Abstract])) OR (Shoulder Hand Syndrome[Title/Abstract])) OR (Shoulder-Hand Syndromes[Title/Abstract])) OR (Algodystrophic Syndrome[Title/Abstract])) OR (Algodystrophy[Title/Abstract])) OR (Algodystrophies[Title/Abstract])) OR (shoulder arm syndrome[Title/Abstract])) OR (sympathetic dystrophy syndrome[Title/Abstract])) OR (sympathetic reflex dystrophy[Title/Abstract])) | 117,054 |
| 7 | "Acupuncture"[Mesh] | 2,015 |
| 8 | ((((((((((((((((((((((((((((((acupuncture[Title/Abstract]) OR (acupoint catgut embedding[Title/Abstract])) OR (point embedding[Title/Abstract])) OR (Acupotomies[Title/Abstract])) OR (Acupotomy[Title/Abstract])) OR (acupuncture therapy[Title/Abstract])) OR (Acupuncture Treatment*[Title/Abstract])) OR (Acupuncture* Auricular[Title/Abstract])) OR (Acupunctures[Title/Abstract])) OR (auriculo acupuncture[Title/Abstract])) OR (auriculotherapy[Title/Abstract])) OR (centro-square needles[Title/Abstract])) OR (Dry Needling[Title/Abstract])) OR (Ear Acupuncture*[Title/Abstract])) OR (earlobe acupuncture[Title/Abstract])) OR (electric* acupuncture[Title/Abstract])) OR (electrical acupoint stimulation[Title/Abstract])) OR (electro* acupuncture[Title/Abstract])) OR (electroacupuncture[Title/Abstract])) OR (fire acupuncture[Title/Abstract])) OR (fire needle[Title/Abstract])) OR (Moxabustion[Title/Abstract])) OR (Moxibustion[Title/Abstract])) OR (needle knife[Title/Abstract])) OR (Pharmacoacupuncture Therapy[Title/Abstract])) OR (Pharmacoacupuncture Treatment[Title/Abstract])) OR (Pharmacopuncture[Title/Abstract])) OR (warm acupuncture[Title/Abstract])) OR (Warming Needle Moxibustion[Title/Abstract])) OR (wrist-ankle acupuncture[Title/Abstract])) OR (scalp acupuncture[Title/Abstract]) | 33,496 |
| 9 | ("Acupuncture"[Mesh]) OR (((((((((((((((((((((((((((((((acupuncture[Title/Abstract]) OR (acupoint catgut embedding[Title/Abstract])) OR (point embedding[Title/Abstract])) OR (Acupotomies[Title/Abstract])) OR (Acupotomy[Title/Abstract])) OR (acupuncture therapy[Title/Abstract])) OR (Acupuncture Treatment*[Title/Abstract])) OR (Acupuncture* Auricular[Title/Abstract])) OR (Acupunctures[Title/Abstract])) OR (auriculo acupuncture[Title/Abstract])) OR (auriculotherapy[Title/Abstract])) OR (centro-square needles[Title/Abstract])) OR (Dry Needling[Title/Abstract])) OR (Ear Acupuncture*[Title/Abstract])) OR (earlobe acupuncture[Title/Abstract])) OR (electric* acupuncture[Title/Abstract])) OR (electrical acupoint stimulation[Title/Abstract])) OR (electro* acupuncture[Title/Abstract])) OR (electroacupuncture[Title/Abstract])) OR (fire acupuncture[Title/Abstract])) OR (fire needle[Title/Abstract])) OR (Moxabustion[Title/Abstract])) OR (Moxibustion[Title/Abstract])) OR (needle knife[Title/Abstract])) OR (Pharmacoacupuncture Therapy[Title/Abstract])) OR (Pharmacoacupuncture Treatment[Title/Abstract])) OR (Pharmacopuncture[Title/Abstract])) OR (warm acupuncture[Title/Abstract])) OR (Warming Needle Moxibustion[Title/Abstract])) OR (wrist-ankle acupuncture[Title/Abstract])) OR (scalp acupuncture[Title/Abstract])) | 33,717 |
| 10 | (("Reflex Sympathetic Dystrophy"[Mesh]) AND (("Reflex Sympathetic Dystrophy"[Mesh]) OR ((((((((((((((((((((((((Reflex Sympathetic Dystrophy[Title/Abstract]) OR (complex regional pain syndrome type I[Title/Abstract])) OR (Reflex Sympathetic Dystrophies[Title/Abstract])) OR (Sudek Atrophy[Title/Abstract])) OR (Sympathetic Reflex Dystrophia[Title/Abstract])) OR (Sympathetic Reflex Dystrophias[Title/Abstract])) OR (Type I Complex Regional Pain Syndrome[Title/Abstract])) OR (Sudek's Atrophy[Title/Abstract])) OR (Sudek's Atrophies[Title/Abstract])) OR (Sudeks Atrophy[Title/Abstract])) OR (Sudeks Atrophy[Title/Abstract])) OR (CRPS Type Is[Title/Abstract])) OR (Reflex Sympathetic Dystrophy Syndrome[Title/Abstract])) OR (Cervical Sympathetic Dystrophy[Title/Abstract])) OR (Cervical Sympathetic Dystrophies[Title/Abstract])) OR (Shoulder-Hand Syndrome[Title/Abstract])) OR (Shoulder Hand Syndrome[Title/Abstract])) OR (Shoulder-Hand Syndromes[Title/Abstract])) OR (Algodystrophic Syndrome[Title/Abstract])) OR (Algodystrophy[Title/Abstract])) OR (Algodystrophies[Title/Abstract])) OR (shoulder arm syndrome[Title/Abstract])) OR (sympathetic dystrophy syndrome[Title/Abstract])) OR (sympathetic reflex dystrophy[Title/Abstract])))) AND (("Acupuncture"[Mesh]) OR (((((((((((((((((((((((((((((((acupuncture[Title/Abstract]) OR (acupoint catgut embedding[Title/Abstract])) OR (point embedding[Title/Abstract])) OR (Acupotomies[Title/Abstract])) OR (Acupotomy[Title/Abstract])) OR (acupuncture therapy[Title/Abstract])) OR (Acupuncture Treatment*[Title/Abstract])) OR (Acupuncture* Auricular[Title/Abstract])) OR (Acupunctures[Title/Abstract])) OR (auriculo acupuncture[Title/Abstract])) OR (auriculotherapy[Title/Abstract])) OR (centro-square needles[Title/Abstract])) OR (Dry Needling[Title/Abstract])) OR (Ear Acupuncture*[Title/Abstract])) OR (earlobe acupuncture[Title/Abstract])) OR (electric* acupuncture[Title/Abstract])) OR (electrical acupoint stimulation[Title/Abstract])) OR (electro* acupuncture[Title/Abstract])) OR (electroacupuncture[Title/Abstract])) OR (fire acupuncture[Title/Abstract])) OR (fire needle[Title/Abstract])) OR (Moxabustion[Title/Abstract])) OR (Moxibustion[Title/Abstract])) OR (needle knife[Title/Abstract])) OR (Pharmacoacupuncture Therapy[Title/Abstract])) OR (Pharmacoacupuncture Treatment[Title/Abstract])) OR (Pharmacopuncture[Title/Abstract])) OR (warm acupuncture[Title/Abstract])) OR (Warming Needle Moxibustion[Title/Abstract])) OR (wrist-ankle acupuncture[Title/Abstract])) OR (scalp acupuncture[Title/Abstract]))) | 47 |

**2.Cochrane**

| Search number | Query | Results |
| --- | --- | --- |
| #1 | MeSH descriptor: [Stroke] explode all trees | 14,225 |
| #2 | (Stroke):ti,ab,kw OR (Strokes):ti,ab,kw OR (Cerebrovascular Accident):ti,ab,kw OR (Cerebrovascular Accidents):ti,ab,kw OR (Cerebrovascular Apoplexy):ti,ab,kw | 69,774 |
| #3 | (Brain Vascular Accident):ti,ab,kw OR (Brain Vascular Accidents):ti,ab,kw OR (Apoplexy):ti,ab,kw | 901 |
| #4 | #1 OR #2 OR #3 | 70,491 |
| #5 | MeSH descriptor: [Reflex Sympathetic Dystrophy] explode all trees | 259 |
| #6 | (complex regional pain syndrome type I):ti,ab,kw OR (Reflex Sympathetic Dystrophies):ti,ab,kw OR (Sudek Atrophy):ti,ab,kw OR (Sympathetic Reflex Dystrophia):ti,ab,kw OR (Sympathetic Reflex Dystrophias):ti,ab,kw | 230 |
| #7 | (CRPS Type I):ti,ab,kw OR (CRPS Type Is):ti,ab,kw OR (Reflex Sympathetic Dystrophy Syndrome):ti,ab,kw OR (Cervical Sympathetic Dystrophy):ti,ab,kw OR (Cervical Sympathetic Dystrophies):ti,ab,kw | 343 |
| #8 | (Shoulder-Hand Syndrome):ti,ab,kw OR (Shoulder Hand Syndrome):ti,ab,kw OR (Shoulder-Hand Syndromes):ti,ab,kw OR (Algodystrophic Syndrome):ti,ab,kw OR (Algodystrophy):ti,ab,kw | 545 |
| #9 | (Algodystrophies):ti,ab,kw OR (shoulder arm syndrome):ti,ab,kw OR (sympathetic dystrophy syndrome):ti,ab,kw OR (sympathetic reflex dystrophy):ti,ab,kw | 875 |
| #10 | #5 OR #6 OR #7 OR #8 OR #9 | 1,189 |
| #11 | MeSH descriptor: [Acupuncture] explode all trees | 199 |
| #12 | (acupuncture):ti,ab,kw OR (acupoint catgut embedding):ti,ab,kw OR (point embedding):ti,ab,kw OR (Acupotomies):ti,ab,kw OR (Acupotomy):ti,ab,kw | 18,084 |
| #13 | (acupuncture therapy):ti,ab,kw OR (Acupuncture Treatment*):ti,ab,kw OR (Acupuncture* Auricular):ti,ab,kw OR (Acupunctures):ti,ab,kw OR (auriculo acupuncture):ti,ab,kw | 14,426 |
| #14 | (auriculotherapy):ti,ab,kw OR (centro-square needles):ti,ab,kw OR (Dry Needling):ti,ab,kw OR (Ear Acupuncture*):ti,ab,kw OR (earlobe acupuncture):ti,ab,kw | 1,946 |
| #15 | (electric* acupuncture):ti,ab,kw OR (electrical acupoint stimulation):ti,ab,kw OR (electro* acupuncture):ti,ab,kw OR (electroacupuncture):ti,ab,kw OR (fire acupuncture):ti,ab,kw | 5,338 |
| #16 | (fire needle):ti,ab,kw OR (Moxabustion):ti,ab,kw OR (Moxibustion):ti,ab,kw OR (needle knife):ti,ab,kw OR (Pharmacoacupuncture Therapy):ti,ab,kw | 2,635 |
| #17 | (Pharmacoacupuncture Treatment):ti,ab,kw OR (Pharmacopuncture):ti,ab,kw OR (warm acupuncture):ti,ab,kw OR (Warming Needle Moxibustion):ti,ab,kw OR (wrist-ankle acupuncture):ti,ab,kw | 462 |
| #18 | (scalp acupuncture):ti,ab,kw | 493 |
| #19 | #11 OR #12 OR #13 OR #14 OR #15 OR #16 OR #17 OR #18 | 21,509 |
| #20 | #4 AND #10 AND #19 | 50 |

**3.Embase**

| Search number | Query | Results |
| --- | --- | --- |
| #1 | 'acute ischemic stroke'/exp | 7,378 |
| #2 | 'acute ischemic stroke':ab,ti OR stroke:ab,ti OR strokes:ab,ti OR 'cerebrovascular accident':ab,ti OR 'cerebrovascular accidents':ab,ti OR 'cerebrovascular apoplexy':ab,ti OR 'brain vascular accident':ab,ti OR 'brain vascular accidents':ab,ti OR apoplexy:ab,ti | 496,384 |
| #3 | #1 OR #2 | 496,772 |
| #4 | 'complex regional pain syndrome type i'/exp | 6,035 |
| #5 | 'reflex sympathetic dystrophy':ab,ti OR 'complex regional pain syndrome type i':ab,ti OR 'reflex sympathetic dystrophies':ab,ti OR 'sudek atrophy':ab,ti OR 'sympathetic reflex dystrophia':ab,ti OR 'sympathetic reflex dystrophias':ab,ti OR 'type i complex regional pain syndrome':ab,ti OR 'sudeks atrophies':ab,ti OR 'sudeks atrophy':ab,ti OR 'crps type i':ab,ti OR 'crps type is':ab,ti OR 'reflex sympathetic dystrophy syndrome':ab,ti OR 'cervical sympathetic dystrophy':ab,ti OR 'cervical sympathetic dystrophies':ab,ti OR 'shoulder-hand syndrome':ab,ti OR 'shoulder hand syndrome':ab,ti OR 'shoulder-hand syndromes':ab,ti OR 'algodystrophic syndrome':ab,ti OR algodystrophy:ab,ti OR algodystrophies:ab,ti OR 'shoulder arm syndrome':ab,ti OR 'sympathetic dystrophy syndrome':ab,ti OR 'sympathetic reflex dystrophy':ab,ti | 3,904 |
| #6 | #4 OR #5 | 7,101 |
| #7 | 'acupuncture'/exp | 56,034 |
| #8 | acupuncture:ab,ti OR 'acupoint catgut embedding':ab,ti OR 'point embedding':ab,ti OR acupotomies:ab,ti OR acupotomy:ab,ti OR 'acupuncture therapy':ab,ti OR 'acupuncture treatment*':ab,ti OR 'acupuncture* auricular':ab,ti OR acupunctures:ab,ti OR 'auriculo acupuncture':ab,ti OR auriculotherapy:ab,ti OR 'centro-square needles':ab,ti OR 'ear acupuncture*':ab,ti OR 'earlobe acupuncture':ab,ti OR 'electrical acupoint stimulation':ab,ti OR 'electro* acupuncture':ab,ti OR electroacupuncture:ab,ti OR 'fire acupuncture':ab,ti OR 'fire needle':ab,ti OR moxabustion:ab,ti OR moxibustion:ab,ti OR 'needle knife':ab,ti OR 'pharmacoacupuncture therapy':ab,ti OR 'pharmacoacupuncture treatment':ab,ti OR pharmacopuncture:ab,ti OR 'warm acupuncture':ab,ti OR 'warming needle moxibustion':ab,ti OR 'wrist-ankle acupuncture':ab,ti OR 'scalp acupuncture':ab,ti | 45,682 |
| #9 | #7 OR #8 | 62,064 |
| #10 | #3 AND #6 AND #9 | 43 |

**4.Web of science**

| Search number | Query | Results |
| --- | --- | --- |
| 1 | Stroke (Topic) OR Strokes (Topic) OR Cerebrovascular Accident (Topic) OR Cerebrovascular Accidents (Topic) OR Cerebrovascular Apoplexy (Topic) OR Brain Vascular Accident (Topic) OR Brain Vascular Accidents (Topic) OR Apoplexy (Topic) | 436,609 |
| 2 | Reflex Sympathetic Dystrophy (Topic) OR complex regional pain syndrome type I (Topic) OR Reflex Sympathetic Dystrophies (Topic) OR Sudek Atrophy (Topic) OR Sympathetic Reflex Dystrophia (Topic) OR Sympathetic Reflex Dystrophias (Topic) OR Type I Complex Regional Pain Syndrome (Topic) OR Sudek's Atrophy (Topic) OR Sudek's Atrophies (Topic) OR Sudeks Atrophy (Topic) OR Sudeks Atrophy (Topic) OR CRPS Type Is (Topic) OR Reflex Sympathetic Dystrophy Syndrome (Topic) OR Cervical Sympathetic Dystrophy (Topic) OR Cervical Sympathetic Dystrophies (Topic) OR Shoulder-Hand Syndrome (Topic) OR Shoulder Hand Syndrome (Topic) OR Shoulder-Hand Syndromes (Topic) OR Algodystrophic Syndrome (Topic) OR Algodystrophy (Topic) OR Algodystrophies (Topic) OR shoulder arm syndrome (Topic) OR sympathetic dystrophy syndrome (Topic) OR sympathetic reflex dystrophy (Topic) | 6,245 |
| 3 | acupuncture (Topic) OR acupoint catgut embedding (Topic) OR point embedding (Topic) OR Acupotomies (Topic) OR Acupotomy (Topic) OR acupuncture therapy (Topic) OR Acupuncture Treatment* (Topic) OR Acupuncture* Auricular (Topic) OR Acupunctures (Topic) OR auriculo acupuncture (Topic) OR auriculotherapy (Topic) OR centro-square needles (Topic) OR Dry Needling (Topic) OR Dry Needling (Topic) OR Dry Needling (Topic) OR electric* acupuncture (Topic) OR electrical acupoint stimulation (Topic) OR electro* acupuncture (Topic) OR electroacupuncture (Topic) OR fire acupuncture (Topic) OR fire needle (Topic) OR Moxabustion (Topic) OR Moxibustion (Topic) OR needle knife (Topic) OR Pharmacoacupuncture Therapy (Topic) OR Pharmacoacupuncture Treatment (Topic) OR Pharmacopuncture (Topic) OR warm acupuncture (Topic) OR Warming Needle Moxibustion (Topic) OR wrist-ankle acupuncture (Topic) OR wrist-ankle acupuncture (Topic) | 78,164 |
| 4 | #1 AND #2 AND #3 | 22 |

# Table S2 Literature for full reading

| No | First Author | Literature name | Was it included? | Reason for exclusion |
| --- | --- | --- | --- | --- |
| 1 | Ying Wang | Observation on Therapeutic Effects of Different Acupoint Selection Methods Combined with Systemati cal Rehabilitation on Shoulder-Hand Syndrome After Apoplexy | Yes |  |
| 2 | Yongqiang He | Immediate Effect Study and fMRI Study of Electroacupuncture on Scalp Acupuncture Treatment of Shoulder-Hand Syndrome after Stroke | Yes |  |
| 3 | Xinru Li | Clinical observation on warm acupuncture and moxibustion for trigger points in patients with stage Ⅰ shoulder-hand syndrome after stroke | Yes |  |
| 4 | Juan Yang | Curative effect observation of fire needle on post-stroke shoulder-hand syndrome in early stage | Yes |  |
| 5 | Qin Xie | Effects of Jin's three-needle acupuncture therapy combined with rehabilitation training in patients with shoulder-hand syndrome after stroke | Yes |  |
| 6 | Xiaoqiong Hua | Clinical study on treatment of qi deficiency and blood stasis syndrome of shoulder-hand syndrome after stroke with wax-mud moxibustion | Yes |  |
| 7 | Liang Zhou | Effects of Acupuncture from Tiaokou to Chenshan and Exercise on Shoulder-hand Syndrome at Stage I after Stroke | Yes |  |
| 8 | Feilin Ni | Effect of Warm-unblocking Therapy Using Moving Cupping and Acupoints Moxibustion in Stroke Patients with Shoulder-hand Syndrome | Yes |  |
| 9 | Qingbo Ju | Clinical Research on the Treatment of Shoulder Hand Syndrome after Stroke by Eye-acupuncture Cooperated with Ironing Analgesia | Yes |  |
| 10 | Wenjun Nong | Effect of Silver Needle with Systematical Rehabilitation on Shoulder-hand Syndrome after Stroke | Yes |  |
| 11 | Yimo Feng | Acupuncture combined with infrared acupoint irradiation and rehabilitation treatment of 126 cases of clinical observation on shoulder hand syndrome after stroke | Yes |  |
| 12 | Xianping Huang | Effect of Acupuncture Combined with Rehabilitation Exercise on Fugl-Meyer Score and Quality of Life in Patients with Shoulder-Hand Syndrome after Stroke | Yes |  |
| 13 | Ranwei Li | Effect of the contralateral needling therapy on post-stroke shoulder-hand syndrome | Yes |  |
| 14 | Lijuan Cao | Analysis of the clinical effect on post-stroke shoulder hand syndrome stage Ⅰ treated with the along-meridian trochar acupuncture therapy | Yes |  |
| 15 | Ronghua Zhu | Treatment of Stroke Patients with Shoulder-wrist Syndrome by Acupoint Catgut Embedding and Surface Electromyogram Biofeedback Therapy | Yes |  |
| 16 | Xiongjie Chen | Clinical Efficacy on Post-stroke Shoulder-hand Syndrome (Stage 1) Treated byElectro-acupuncture at Dorsal Root of Auric le | Yes |  |
| 17 | Zouqin Huang | Clinical Observation of Acupuncture plus Medicine and Function Training for Post-stroke Shoulder-hand Syndrome | Yes |  |
| 18 | Miaojun Lin | Clinical observation on the treatment of post-stroke shoulder-hand syndrome by Fuyang buried thread method | Yes |  |
| 19 | Liwen Xue | Clinical observation on penetration needling combined with electroacupuncture for treatment of poststroke shoulder-hand syndrome | Yes |  |
| 20 | Ruiqing Li | Clinical observation on wrist-ankle acupuncture for shoulder-hand syndrome phase Ⅰ after stroke | Yes |  |
| 21 | Yanjie Shang | Clinical study on acupuncture combined with rehabilitation therapy for treatment of poststroke shoulder-hand syndrome | Yes |  |
| 22 | Jie Zhan | Effect of abdominal acupuncture combined with routine rehabilitation training on shoulder-hand syndrome after stroke: A randomized controlled trial | Yes |  |
| 23 | Fanying Meng | Effect of Warm Acupuncture Stimulation of Waiguan (TE 5) on Post-stroke Shoulder-hand Syndrome | Yes |  |
| 24 | Xiaoli Tang | Muscle regions of meridians warm needling method plus pricking Jing-Well points for blood-letting in the treatment of shoulder-hand syndrome after stroke | Yes |  |
| 25 | Jun Wang | The efficacy of floating stabbing combined with rehabilitation training for the treatment of shoulder pain in shoulder-hand syndrome after stroke | Yes |  |
| 26 | Jinbiao Hong | Observation on therapeutic effect of opposing needling for treatment of poststroke shoulder-hand syndrome | Yes |  |
| 27 | Qian Zhang | Observations on the Efficacy of Along-meridian Distal Point Selection in Treating Post-cerebral Infarction Shoulder-hand Syndrome | Yes |  |
| 28 | Bing Yan | Observations on the Efficacy of Jin’s Three Needles plus Twelve Jing-Well Points in Treating Post-stroke Shoulder-hand Syndrome | Yes |  |
| 29 | Tongbo Jiang | Observations on the Efficacy of Superficial Needling plus External Washing with Chinese Herbal Medicine in Treating Post-stroke Shoulder-hand Syndrome | Yes |  |
| 30 | Chunshui Huang | Penetration acupuncture at Baxie(EX-UE 9)combined with rehabilitation for swelling hand of post-stroke shoulder-hand syndrome | Yes |  |
| 31 | Zhaohui Zhou | Post-stroke shoulder-hand syndrome treated with floating-needle therapy combined with rehabilitation training: a randomized controlled trial | Yes |  |
| 32 | Wenrong Wan | Acupuncture combined with rehabilitation for post-stroke shoulder-hand syndrome: a randomized controlled study | Yes |  |
| 33 | Bingfeng Xing | Short-term and long-term efficacy of PGLA thread-embedding therapy in treatment of stageⅠ post-stroke shoulder-hand syndrome | Yes |  |
| 34 | Ning Li | Therapeutic effect of acupuncture and massage for shoulder-hand syndrome in hemiplegia patients: a clinical two-center randomized controlled trial | Yes |  |
| 35 | Jingchun Yin | Therapeutic Observation of Acupuncture at the Interiorly-exteriorly Related Meridians plus Rehabilitation Training for Post-stroke Shoulder-hand Syndrome | Yes |  |
| 36 | Jingjun Xie | Therapeutic Observation of Electroacupuncture plus Rehabilitation for Post-stroke Shoulder-hand Syndrome | Yes |  |
| 37 | Sen Gao | WANG Ju-yi's meridian diagnosis method combined with Bobath rehabilitation training for post-stroke shoulder-hand syndrome type Ⅰ | Yes |  |
| 38 | Zhihong Zou | Effect of White Tiger Shaking Head Needling Combined with Rehabilitation Training on Pain and Upper Limb Motor Function in the Treatment of SHSAS | Yes |  |
| 39 | Xueping Yu | Therapeutic Effect of Baihu Yaotou Needling on post-stroke Shoulder-hand Syndrome. Journal of Clinical Acupuncture and Moxibustion | Yes |  |
| 40 | Shurong Wang | Application Research of Spoon Needle Acupressure Combined with Shoulder Triple Needling in Treatment of Post-Stroke SHS | Yes |  |
| 41 | Mingming Wang | Clinical Observation of Electro-acupuncture Combined with Medicated Cake Moxibustion Treating Post-Stroke Shoulder-Hand Syndrome of Phase Ⅰ | Yes |  |
| 42 | Weidong Yang | Therapeutic Observation of Electroacupuncture plus Mirror Therapy for Shoulder-hand Syndrome Stage Ⅰ after Cerebral Stroke | Yes |  |
| 43 | Yang Wang | Efficacy Observation of Surrounding Electro-Acupuncture Combined with TCM Fumigation on Post-Stroke Shoulder-Hand Syndrome | Yes |  |
| 44 | Shengsan He | Evaluation of Abdominal Acupuncture and Rehabilitation Treatment for Shoulder-hand Syndrome (Period Ⅰ) after Stroke | Yes |  |
| 45 | Jingchang Huang | Clinical Observations on Fire Needle Acupuncture at Muscular Attachments for the Treatment of Post-stroke Shoulder-hand Syndrome | Yes |  |
| 46 | Youhua Zeng | Clinical Observation of Ling Gui Ba Fa for Post-stroke Shoulder-hand Syndrome Stage Ⅰ | Yes |  |
| 47 | Ting Zhang | Clinical Effects of Balanced Acupuncture Combined with Bobath Therapy on Post-Stroke Shoulder-Hand Syndrome | Yes |  |
| 48 | Fei Huang | Clinical Study of Thumb-Tack Needling Combined with Electro-Acupuncture and Rehabilitation Treating Post-Stoke Shoulder-Hand Syndrome | Yes |  |
| 49 | Guoqi Chen | Clinical Observation of Point-to-Point Scalp Acupuncture Combined with Body Acupuncture in the Treatment of Shoulder-Hand Syndrome after Stroke | Yes |  |
| 50 | Xin Tong | Therapeutic Observation of Early-stage Shoulder-hand Syndrome after Ischemic Stroke Treated with Acupuncture at Distal Points plus Exercise | Yes |  |
| 51 | Qin Xie | Effect of Jin's three-needle therapy (JTNT) on Shoulder-Hand Syndrome after Stroke | No | The same RCT, repeatedly published studies with different outcomes or populations |
| 52 | Wenjun Nong | Clinical study on silver needle combined with rehabilitation system for shoulder-hand syndrome after stroke | No | The same RCT, repeatedly published studies with different outcomes or populations |
| 53 | Lijuan Cao | Effect of trocar acupuncture along meridians on main abnormal meridian states in patients with shoulder-hand syndrome after apoplexy | No | The same RCT, repeatedly published studies with different outcomes or populations |
| 54 | Yang Wang | Clinical observation on the surrounding electroacupuncture in treatment of shoulder-hand syndrome after stroke | No | The same RCT, repeatedly published studies with different outcomes or populations |
| 55 | Yan Shao | Randomized controlled clinical trial of eye-acupuncture cooperated with ironing analgesia in the treatment of shoulder-hand syndrome after stroke | No | The same RCT, repeatedly published studies with different outcomes or populations |
| 56 | Junlong Dai | Effect of stellate ganglion block on patients with shoulder-hand syndrome after cerebral infarction | No | The same RCT, repeatedly published studies with different outcomes or populations |
| 57 | Bin Zhu | Observation on therapeutic effect of binding therapy combined with acupuncture on shoulder-hand syndrome | No | Full text of conference abstracts published without peer review |
| 58 | Jun Xu | Comparative analysis of therapeutic effects of acupotomy and acupuncture in the treatment of shoulder-hand syndrome after stroke | No | Full text of conference abstracts published without peer review |
| 59 | Yudong Tao | Effect of acupuncture point injection and rehabilitation technology on upper limb function and living ability of shoulder-hand syndrome after stroke | No | Full text of conference abstracts published without peer review |
| 60 | Jia Shi | Clinical observation on scalp acupuncture combined with body acupuncture in the treatment of shoulder-hand syndrome after stroke | No | Full text of conference abstracts published without peer review |
| 61 | Lietao He | Effect of acupuncture based on syndrome differentiation of Zang-Fu organs and meridians on quality of life of patients with hemiplegic shoulder pain after stroke | No | No interest outcome indicators |
| 62 | Xiangyang Zhai | Observation on therapeutic effect of surrounding needling on shoulder-hand syndrome after stroke | No | No interest outcome indicators |
| 63 | Xin Ren | Treatment of shoulder-hand syndrome in stage Ⅰ after cerebral hemorrhage with traditional Chinese medicine, acupuncture and rehabilitation | No | No interest outcome indicators |
| 64 | Shukai Han | Analysis of therapeutic effect of acupuncture combined with medicine on patients with shoulder-hand syndrome after stroke | No | Seriously unreasonable in design |
| 65 | Suoling Lu | Efficacy and nursing care of acupuncture combined with fumigation of traditional Chinese medicine in the treatment of shoulder-hand syndrome after stroke | No | Seriously unreasonable in design |
| 66 | Yiting Chai | Clinical analysis of acupuncture combined with oral administration of traditional Chinese medicine in the treatment of shoulder-hand syndrome after stroke | No | Seriously unreasonable in design |

# TableS3 Intervention methods - brief

| Treat 1 (rehabilitation) | It includes routine rehabilitation treatments such as correct positioning, facilitation training, Bobath technique, PNF technique, Rood's technique, active and passive full range of motion (ROM) of limbs and joints, reduction of muscle tension, suppression of abnormal patterns, and balance training |  |
| --- | --- | --- |
| Treat 2 (rehabilitation + western medicine) | Western medicine treatment is adopted on the basis of rehabilitation treatments, such as oral administration of western medicines to relieve pain, improve cerebral blood circulation, nourish nerves, regulate blood pressure, regulate blood glucose and regulate blood lipid and other symptomatic and supportive treatments of western medicine. |  |
| Treat 3 (routine acupuncture) | Acupuncture treatment is performed on acupoints of affected limbs. |  |
| Treat 4 (rehabilitation + acupuncture) | Acupuncture treatment is adopted on the basis of rehabilitation treatment, including routine acupuncture at acupoints of affected limbs, trigger point acupuncture, penetration needling at Baxie, etc. |  |
| Treat 5 (western medicine + rehabilitation + acupuncture) | Combined treatment of western medicine, rehabilitation and acupuncture |  |
| Treat 6 (electroacupuncture) | Electroacupuncture is a therapy for treating diseases by applying the electroacupuncture therapy device to deliver a feeble current close to the bioelectrical current of the human body on the basis of obtaining the qi by filiform needle acupuncture. Electroacupuncture combines the filiform needle with electrical stimulation, which not only reduces the workload of needle operation but also improves the therapeutic effect of the filiform needle, expands the therapeutic range of the filiform needle, and accurately controls the amount of stimulation. Different stimulation wave combinations can be obtained through frequency adjustment, and electrical stimulation at different frequencies can promote the release of different central neurotransmitters. For example, 2 Hz and 100 Hz electrical stimulation increases the contents of enkephalin and endorphin in cerebrospinal fluid and of dynorphin respectively; 2/100 Hz alternating dilatational waves enable endorphin and dynorphin to be released simultaneously. Electroacupuncture has basically the same scope of application as filiform needle acupuncture, with the functions of analgesia, sedation, improvement of blood circulation, adjustment of muscular tension, etc. |  |
| Treat 7 (warm acupuncture) | Warm acupuncture refers to the method of inserting the moxa or the moxa stick in the needle handle during retention of the filiform needle to conduct acupuncture and moxibustion simultaneously, also known as needle warming moxibustion. During the operation, the filiform needle is inserted into the acupoint. After obtaining the qi and employing a proper reinforcing and reducing method, the needle is left at an appropriate depth and the moxa bag is wrapped around the needle end. Alternatively, the moxa stick of 2 - 3 cm in length is directly inserted into the needle handle and ignited for acupuncture. After the moxa or the moxa stick is burned out, ashes will be cleaned up, and the needle will be removed. Care should be taken to prevent moxa fire from falling on the skin, causing skin burns. This method is a combination application of acupuncture and moxibustion and is applicable to diseases in need of needle retention for which moxibustion is suitable. |  |
| Treat 8 (fire needling) | Fire needling is a method for treating diseases by rapidly piercing the burning needle of a specially-made acupuncture apparatus into a certain body part for local scorching stimulation. It is historically known as "red-hot needling", and also known as "wei-needle" and "fan-needle". Acupuncture apparatus for fire needling is generally made of metal materials that can withstand high temperatures, is not easily folded under high temperatures, has high hardness, and is harmless to the human body. Operation method: 1. Needle burning: Burn the needle, with one hand holding an ignited alcohol light and the other hand holding the needle. The needle should be burned at a position close to the treatment site. Generally, the needle is burned first followed by the needle tip. The degree of burning can be determined according to the acupuncture depth. If the target site is deep, the needle should be burnt until turning white; if the site is shallow, the needle should be burnt until turning red; if the needle is only used to iron the body surface slightly and slowly, the needle should be burnt until turning slightly red. After the needle is burnt sufficiently, it should be quickly and accurately inserted into the target site and removed rapidly or retained for 5 to 15 minutes before being removed. After completing fire needling, the pinhole should be pressed immediately with a sterile dry cotton ball to reduce pain and prevent bleeding. |  |
| Treat 9 (Jin's three-needle technique) | Jin's three-needle technique is a clinical acupuncture therapy mainly performed with three or three groups of acupoint formulas. This therapy was so named because it was invented by Jin Rui and three or three groups of matching acupoints are selected for each treatment. It features the careful selection of a small number of acupoints, and three or three groups of acupoints usually suffice to treat many clinical diseases, with obvious therapeutic effects. It is indicated for pediatric cerebral palsy, stroke hemiplegia, childhood autism, senile dementia, optic atrophy, insomnia, gout, dizziness, headache, etc. |  |
| Treat 10 (floating needle) | Floating needle therapy is a modern new acupuncture method by which a specially-made disposable floating needle apparatus is used to sweep the subcutaneous superficial fascia around the site of localized pain. During operation, patients are usually required to actively or passively move the affected muscles or joints to make the relevant muscles relax and contract. It is characterized by freedom from pain, quick effect, a wide spectrum of indications, etc. |  |
| Treat 11 (eye acupuncture) | Eye acupuncture therapy is a microneedle technique used to perform acupuncture in specific acupoint areas around the orbit and is characterized by the use of small needles, selection of a small number of acupoints, shallow insertion position, gentle manipulation, simple operation, safety, and freedom from pain. Diseases that can be treated with acupuncture can also be treated with eye acupuncture, especially stroke, headache, dizziness, insomnia, pain, etc. |  |
| Treat 12 (special needle operation and technique) | After completing acupuncture, the operator moves the needle with his or her hand to make the filiform needle move in acupoints. The techniques to increase the amount of stimulation at acupuncture sites include white tiger shaking head needling, Tianzong acupuncture by dragon and tiger fighting needling, balance acupuncture, along-meridian distal point selection therapy, multi-needle superficial needling, surrounding electroacupuncture, penetration needling, etc. | White tiger shaking head needling: It refers to a compound technique of needle operation. After entering the acupoint, the filiform needle is pushed and rotated to the left and then lifted and rotated to the right while being shaken from side to side as if a bell is shaken by hand. The procedure is then repeated six or multiples of six times. It plays the role of promoting the circulation of meridian qi and blood, thus dispersing blood stasis. |
|  |  | Dragon and tiger fighting needling: It is a compound technique of needle operation, by which the filiform needle is twirled repeatedly from side to side in an alternate way during needle operation for the purpose of stimulation. Operation method: After inserting the filiform needle into the acupoint and obtaining the qi, twirl the needle clockwise 9 times, and then twirl the needle counterclockwise 6 times. After repeating the above operation three times, repeat it once at an interval of 5 minutes, with the needle retained for 30 minutes each time for a total of 6 times, until the patient feels that the affected limb is comfortable and warm. When the needle is inserted to a certain depth in the acupoint by this method, it may be entwisted by tissue fibers after being twirled in one direction several times, resulting in a strong needling sensation. The analgesic effect of this method is closely associated with the times and angles of twirling the needle. |
|  |  | Balance acupuncture: It refers to an acupuncture technique by which a specific acupoint on a healthy human limb is selected for acupuncture, after which the needle is moved by the operator by hand and not removed until the distal end of the target limb is subject to conductive discharge with a sense of tingling and swelling, so as to stimulate and mobilize the body's own defense system for self-repair, self-improvement, and self-regulation. |
|  |  | Along-meridian distal point selection therapy: It refers to an acupuncture therapy by selecting Zhongzhu and Houxi at the distal end of the affected limb. During needle retention, the patient is required to carry out flexion, extension, abduction, adduction, external rotation, and internal rotation of the upper limb to the extent that it can be barely tolerated by the patient. |
|  |  | Superficial needling: It refers to shallow acupuncture with the needle without damaging the muscle. |
|  |  | Surround needling: It refers to the method of encircling acupuncture around the area of pain, swelling and pain. |
|  |  | Penetration needling: It is also known as point-through-point or piercing needling, by which a needle is used to penetrate multiple adjacent acupoints or meridians. By this method, a single needle can be used for multiple acupoints, thus simplifying the operation and imposing a stronger sense of needling. The common combinations of penetration needling include acupuncture from Jianyu to Jiquan, Quchi to Shaohai, Hegu to Laogong, and Tiaokou to Chengshan. |
| Treat 13 (other multi-needle acupuncture combination) | It includes silver needle acupuncture, eightfold method of the sacred tortoise, wrist-ankle acupuncture, along-meridian trocar acupuncture, along-meridian distal point acupuncture plus exercise, spoon-like needle acupuncture, thumbtack needle acupuncture, and other acupuncture therapies | The silver needle is a kind of acupuncture apparatus made of 85% silver doped with a small amount of copper and chromium alloy by means of smelting, with a thickness of about 0.8 to 1.1 mm, which is applicable to various human body sites. During use, local anesthesia and disinfection are required. |
|  |  | Eightfold method of the sacred tortoise: It is a method to perform acupuncture on acupoints according to time. Specifically, the principle of eight trigrams is utilized to determine one to two acupoint(s) most suitable for patients from the eight acupoints where the qi and blood of eight extraordinary meridians in the human body are converged and connected, according to the time designated by the heavenly stems and earthly branches by means of mathematical addition and division and the changes of qi-blood opening and closing in the eight acupoints, with the patient's visit time and condition taken into account. |
|  |  | Wrist-ankle acupuncture: It refers to an acupuncture therapy by which subcutaneous acupuncture is performed on wrists and ankles. |
|  |  | Trochar acupuncture: It refers to a new acupuncture method by which a disposable subcutaneous trocar is shallowly inserted subcutaneously mainly for the treatment of pain diseases. Along-meridian trocar acupuncture: It refers to an acupuncture therapy by which the acupoint is selected according to the meridians where the lesion is located. |
|  |  | Along-meridian distal point acupuncture plus exercise: It refers to an acupuncture therapy by which acupoints in different meridians are punctured according to the relationship between the pain site and meridian circulation while moving the patient's affected shoulder. During needle retention, the patient is required to carry out flexion, extension, abduction, adduction, external rotation, and internal rotation of the upper limb to the extent that it can be barely tolerated by the patient. |
|  |  | Spoon-like needle: It is one of the "nine needles" in ancient China. It is 75 to 100 mm long with a blunt and round head and a slightly thick handle and is not indicated for puncture into the skin for the purpose of pushing and pressing of acupoint surface. It can also be connected to a pulse electroacupuncture therapy device to stimulate the corresponding acupoints after being coated with conductive liquid or paste to the extent that it can be barely tolerated by the patient. |
|  |  | Thumbtack needle: It is an intradermal needle shaped like a pushpin, with a flat handle, which is embedded in subcutaneous tissues of corresponding acupoints based on dialectical selection of acupoints, thus being used in shallow acupuncture, and can be retained for a long time to exert sustained stimulation. During needle embedding, patients can press the acupuncture site themselves according to their condition to strengthen the stimulation, thus improving the therapeutic effect. |
| Treat 14 (acupuncture on non-affected limb) | As the circulation of meridians is symmetrical in pair in the human body, and meridians are connected with each other, diseases can be treated by needling acupoints other than those on the affected limb, including needling of the unaffected limb, contralateral acupuncture from Tiaokou to Chengshan plus affected limb movement for hemiplegia, abdominal acupuncture, scalp point penetration needling, contralateral blood-letting and contralateral needling, electroacupuncture of dorsal root of the auricle, contralateral electroacupuncture of anterior/posterior oblique line of vertex-temporal of the affected limb, etc. | Contralateral acupuncture from Tiaokou to Chengshan plus affected limb movement for hemiplegia: It refers to penetration needling of Tiaokou and Chengshan on the unaffected limb in combination with affected limb movement during the operation. |
|  |  | Abdominal acupuncture: It is an acupuncture therapy by which the needle is used to puncture the abdomen to adjust qi movement and yin and yang to strike a dynamic balance between yin and yang under the guidance of the theory of traditional Chinese medicine, thus treating systemic diseases. From the perspective of traditional Chinese medicine, there are many meridians in the human body, functioning as the hub for the systemic delivery of gas and blood. Therefore, the therapeutic effect of this therapy in treating visceral diseases and chronic systemic diseases is significant. |
|  |  | Contralateral blood-letting and contralateral needling: They are two traditional acupuncture methods in ancient China, by which the corresponding acupoints on the right side of the human body are punctured if the left side of the body is affected by a disease, and vice versa. |
|  |  | Scalp acupuncture: It is also known as scalp acupuncture therapy, called scalp acupuncture for short. It was developed on the basis of the Chinese traditional science of acupuncture and moxibustion by utilizing the principle of cerebral cortex functional localization in terms of the corresponding refraction relation on the scalp. Stimulation of this area and the acupoints in this area can affect the related cerebral cortex functions to achieve the purpose of treating diseases.   There are 14 standard lines to be stimulated by scalp acupuncture, including the anterior/posterior oblique line of vertex-temporal. |
| Treat 15 (western medicine + acupuncture + Chinese medicine) | Chinese medicine treatment is adopted on the basis of western medicine and acupuncture treatment |  |
| Treat 16 (rehabilitation + catgut embedding) | Treatment by catgut embedding is adopted on the basis of rehabilitation therapy | Catgut embedding: It is also known as acupoint catgut embedding therapy, by which the absorbable surgical suture is placed into the acupoint to prevent and treat the disease through continuous stimulation of the acupoint by the absorbable suture. Catgut embedding operation method: After local skin disinfection, perform local infiltration anesthesia with lidocaine, take about 1 cm of sterilized absorbable surgical suture, and put it into the front end of a specific trocar, which is then connected with a stylet. Then, secure the acupoint with the thumb and index finger of one hand, and insert the needle with the other hand. After reaching the desired depth, push the stylet to send the suture into the muscular layer or subcutaneous tissue of the acupoint, and then remove the needle. After pressing the pinhole with a sterile dry cotton ball for a moment, wrap it with a sterile dressing to protect the wound for 3 to 5 days. |
| Treat 17 (western medicine + rehabilitation + Chinese medicine) | Chinese medicine treatment is adopted on the basis of western medicine and rehabilitation treatment |  |
| Treat 18 (gangliolysis) | Gangliolysis is performed for the stellate ganglion on the affected side |  |
